# Mechanisms of Sleep/Wake Regulation under Hypodopaminergic State: Insights from MitoPark Mouse Model of Parkinson's Disease

**DOI:** 10.1002/advs.202203170

**Published:** 2022-12-14

**Authors:** Karim Fifel, Masashi Yanagisawa, Tom Deboer

**Affiliations:** ^1^ International Institute for Integrative Sleep Medicine (WPI‐IIIS) University of Tsukuba 1‐1‐1 Tennodai Tsukuba Ibaraki 305–8575 Japan; ^2^ Department of Cell and Chemical Biology Laboratory of Neurophysiology Leiden University Medical Center P.O. Box 9600 Leiden 2300 RC The Netherlands

**Keywords:** dopamine, motivational valence, Parkinson's disease, sleep

## Abstract

Sleep/wake alterations are predominant in neurological and neuropsychiatric disorders involving dopamine dysfunction. Unfortunately, specific, mechanisms‐based therapies for these debilitating sleep problems are currently lacking. The pathophysiological mechanisms of sleep/wake alterations within a hypodopaminergic MitoPark mouse model of Parkinson's disease (PD) are investigated. MitoPark mice replicate most PD‐related sleep alterations, including sleep fragmentation, hypersomnia, and daytime sleepiness. Surprisingly, these alterations are not accounted for by a dysfunction in the circadian or homeostatic regulatory processes of sleep, nor by acute masking effects of light or darkness. Rather, the sleep phenotype is linked with the impairment of instrumental arousal and sleep modulation by behavioral valence. These alterations correlate with changes in high‐theta (8–11.5 Hz) electroencephalogram power density during motivationally‐charged wakefulness. These results demonstrate that sleep/wake alterations induced by dopamine dysfunction are mediated by impaired modulation of sleep by motivational valence and provide translational insights into sleep problems associated with disorders linked to dopamine dysfunction.

## Graphical abstract

What is the pathophysiology of sleep/wake alterations in Parkinson's Disease (PD)? Using MitoPark mouse model of PD, this study highlights the role of motivational valence in shaping sleep/wake cycle and provides translational insights into sleep problems associated with disorders linked to dopamine dysfunction.

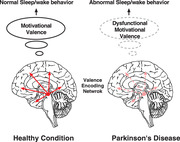

## 1 Introduction

Dysregulations of sleep/wake behavior are core clinical features in virtually all patients suffering from neurological and neuropsychiatric disorders involving dopamine (DA) dysfunction.^[^ ^1^, ^2^, ^3^, ^4^ ^]^ Parkinson's Disease (PD) patients, for example, experience four main sleep symptoms [i.e., REM sleep behavioral disorders (RBD), excessive daytime sleepiness (EDS), insomnia, and sleep/wake fragmentation] that dominate the diagnostic picture and worsen over disease progression.^[^ ^5^, ^6^, ^7^ ^]^ Although the etiology of these sleep alterations is thought to involve complex interaction between multiple factors including intrinsic neuropathology, motor and non‐motor symptoms (NMS), and medical treatments.^[^ ^5^, ^6^, ^7^ ^]^ the underlying neuropathological mechanisms remain poorly understood.

Classical lesional and pharmacological manipulations as well as recent chemo‐ and optogenetic studies have firmly implicated midbrain DA signaling in the modulation of both sleep/wake cycle^[^ ^8^, ^9^, ^10^, ^11^, ^12^, ^13^, ^14^, ^15^, ^16^, ^17^ ^]^ and electroencephalogram (EEG)/local field potential brain rhythms.^[^ ^18^ ^]^ Dysfunctional DA neurotransmission is therefore expected to precipitate qualitative and quantitative alterations of sleep/wake behavior. The pathophysiological mechanisms by which DA dysfunction leads to these sleep/wake dysregulations remain, however, largely unknown.

According to models of sleep regulation,^[^ ^19^, ^20^, ^21^ ^]^ features of sleep/wake behavior are governed by the interaction of homeostatic and circadian processes. The homeostatic process tracks sleep need over wakefulness and adjusts sleep depth accordingly during subsequent sleep, while the circadian process is responsible for the regulation of the timing of sleep and wake episodes within the 24 h day.^[^ ^19^, ^20^, ^21^ ^]^ Two additional factors are known to exert powerful influence on sleep/wake cycle. The first, known as masking, involves a direct and acute (but sustained) impact of light and darkness on sleep and wake centers in the brain.^[^ ^22^, ^23^, ^24^ ^]^ The second is linked to the allostatic modulation of sleep by the saliency of the environment and involves the extent of motivational arousal of the organism.^[^ ^25^ ^]^ A dysfunction of one or a combination of these processes could contribute to pathological sleep/wake behavior.

In the present study, we characterized in detail the nature and extent of sleep/wake alterations in the MitoPark mouse model of PD. These mice faithfully replicate the progressive age‐related neurodegeneration of midbrain DA neurons leading to a hypodopaminergic state and a typical behavioral parkinsonism that closely resembles motor symptoms of PD patients.^[^ ^26^ ^]^ We then investigated the mechanisms of sleep alterations in MitoPark mice by systematically dissecting the potential contribution of each regulatory process to these sleep/wake dysregulations.

Consistent with the wake promoting effect of DA,^[^ ^9^ ^]^ MitoPark mice display profound hypersomnia that was more manifest during the active phase of the light/dark (LD) cycle. Surprisingly, this sleep phenotype was not explained by dysfunctional homeostatic and circadian processes of sleep regulation. Additionally, the masking proprieties of light and darkness on sleep/wake behavior could not account for the sleep alterations in MitoPark mice. Consistent with the role of DA in modulating motivational drive,^[^ ^27^, ^28^, ^29^, ^30^, ^31^, ^32^ ^]^ MitoPark mice displayed a lower threshold of initiating sleep even in highly silent environments. Finally, we discovered a new DA‐mediated modulation of sleep amount by behavioral valence. Importantly, this motivational valence‐related modulation of sleep was independent of the homeostatic regulation of sleep/wake behavior. Collectively, our study provides strong evidence implicating DA‐mediated modulation of motivational valence as a powerful determinant of sleep/wake. This modulation was uncoupled from the classical circadian and homeostatic processes of sleep regulation. Our results advance also new translational insights for sleep/wake alterations associated with several neurological and neuropsychiatric disorders linked to dysfunctional DA signaling.

## 2 Results

### 2.1 Mitopark Mice Recapitulate Most, But Not All, Sleep Phenotypes Experienced by PD Patients

**Figure 1:**
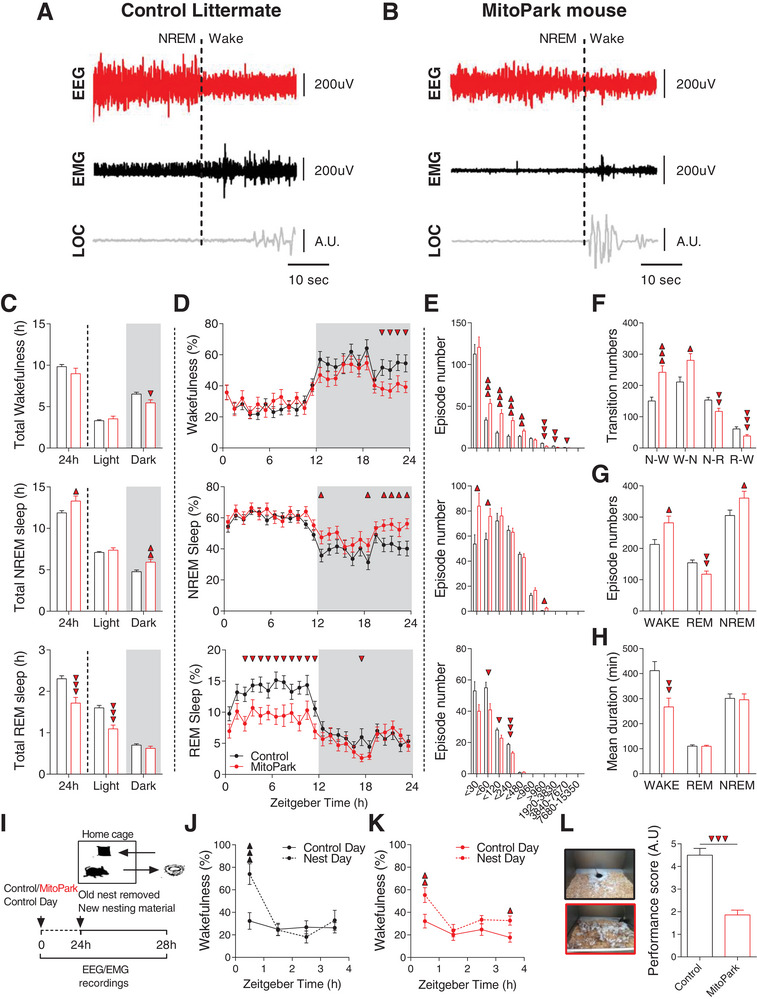
MitoPark mice replicate most of PD‐related sleep alterations. Representative example of a NREM‐to‐wake transition in A) control and B) MitoPark mouse with original EEG, EMG, and locomotor activity traces. C) Mean time and D) hourly percentages spent in wakefulness, NREM, and REM sleep in control (*n* = 19, 10 males) and MitoPark mice (*n* = 19, 12 males). *T*‐test and Two‐way ANOVA, *F*_1,23_ (wake) = 1.21, *F*_1,23_ (NREM) = 0.946, *F*_1,23_ (REM) = 1.783. Sidak's post hoc analysis, ▼ *p* < 0.05, ▼▼ *p* < 0,01, ▼▼▼ *p* < 0,001. E) Number of wake (up), NREM (middle), and REM sleep (down) bouts with different durations during the 24 h day (Two‐way ANOVA, ▼ *p* < 0.05, ▼▼ *p* < 0,01, ▼▼▼ *p* < 0,001). F) Number of all vigilance states transitions is altered in MitoPark mice during LD (Two‐way ANOVA followed by Sidak's post‐hoc correction, ▼ *p* < 0.05, ▼▼ *p* < 0,01, ▼▼▼ *p* < 0,001). G) Episode number of both wake and NREM sleep was increased while the number of REM episodes decreased in MitoPark mice relative to control littermates (Two‐way ANOVA followed by Sidak's post‐hoc correction, ▲ *p* < 0.05, ▼▼ *p* < 0,01). H) MitoPark mice had shorter wake episodes and normal durations of both REM and NREM sleep (Two‐way ANOVA followed by Sidak's post‐hoc correction, ▼▼ *p* < 0,01). I) Schematic diagram depicting nest building behavior experiment. Percentage of time spent awake during the 4 h following the presentation of new nesting material in J) control and K) MitoPark mice (*n* = 19 per each group, two‐way RM ANOVA revealed condition × time interaction for both groups, *F*_1,3_ (control) = 5.687, *p* = 0.001; *F*_1,3_ (MitoPark) = 1.332, *p* < 0.01. Sidak's post‐hoc analysis, ▲ *p* < 0.05, ▲▲ *p* < 0,01, ▲▲▲ *p* < 0,001). L) Left: representative images of control (top) and MitoPark (bottom) mice cages at the end of the 4 h polysomnographic recordings. Right: nesting score representing the amount of nesting material used and final shape of the nest after the 1 h period test (1, poor; 5, good) (Wilcoxon matched‐pairs signed rank test, ▼▼▼ *p* < 0,001). Data represent mean ± sem.

PD patients suffer from five main sleep symptoms, namely; night‐time insomnia, EDS, overall hypersomnia, fragmentation of sleep/wake cycle, and RBD.^[^ ^5^, ^6^ ^]^ Similar to the best animal model of PD (i.e., MPTP‐treated non‐human primates,^[^ ^33^ ^]^ MitoPark mice displayed most of these symptoms (**Figure** **1** and Figure S1, Supporting Information). We performed longitudinal EEG/electromyogram (EMG) recordings under both LD (Figure 1) and DD conditions (Figure S1, Supporting Information). MitoPark mice showed comparable features of raw EEG/EMG signals relative to their control littermates (Figure 1A,B). MitoPark mice exhibited reduced time spent in wakefulness and increased time spent in non‐rapid eye movement (NREM) sleep (Figure 1C,D). Resembling EDS in PD patients, this hypersomnia was mainly manifest during the dark phase of the LD cycle (Figure 1C,D). The sleep/wake cycle was more fragmented in MitoPark mice compared to littermate controls as evidenced by a significant increase in both the number of short episodes (<8 min long, Figure 1E) and in the overall episode number of both wake and NREM sleep (Figure 1G). Consistently, the number of NREM‐wake and wake‐NREM transitions increased in MitoPark mice (Figure 1F). Unlike NREM sleep, but consistent with the sleep phenotype in PD patients,^[^ ^5^, ^6^ ^]^ REM sleep was significantly decreased in MitoPark mice (Figure 1C,D). This decrease is explained by a significant decrease in the number of REM sleep episodes (Figure 1E,G) without an impact on their mean duration (Figure 1H). The only vigilance state that showed a significant decrease in its mean episode duration was wakefulness (Figure 1H).

The total amount of NREM sleep during the light phase was not affected in MitoPark mice (Figure 1C,D) showing therefore that this mouse model does not replicate the reduced NREM sleep amount reported during night‐time in PD patients. Similarly, we did not observe any evidence of RBD behavior in MitoPark mice. We also examined sleep/wake architecture under constant darkness (DD) and found similar alterations in MitoPark mice as shown under LD cycle (Figure S1, Supporting Information). MitoPark mice replicate also the progressive deterioration of motor symptoms^[^ ^26^ ^]^ and in PD patients, sleep alterations worsen over disease progression.^[^ ^65^ ^]^ To assess if that is also the case for MitoPark mice, we compared sleep architecture in early stages (4.8 ± 0.13 months old) and advanced stages (6.4 ± 0.23 months old) of parkinsonism in MitoPark mice (Figures S2 and S3, Supporting Information). The hypersomnia in MitoPark mice was evident mainly in the late half of the dark phase in the early stage (Figure S2, Supporting Information) while in the advanced stage, MitoPark mice showed the hypersomnia during both early and late hours of the dark phase (Figure S3, Supporting Information). REM sleep was similarly reduced during the light phase in both groups (Figures S2 and S3, Supporting Information).

We finally examined the performances of MitoPark mice in nest‐building behavior. To this end, we introduced new nesting material to animal's home cage at the start of the light phase (when nesting behavior normally takes place) and recorded EEG/EMG signals for the subsequent 4 h (Figure 1I). Both control and MitoPark mice increased the percentage of wake (though to a lesser extent in MitoPark mice) during the first hour of contact with the new nesting material relative to a control day (Figure 1J,K). Consistent with a previous study implicating ventral tegmental area (VTA) DA neurons in nesting behavior,^[^ ^10^ ^]^ MitoPark mice had bad performances as evidenced by significantly low nest building scores achieved, compared to controls (Figure 1L). Collectively, our results demonstrate that DA loss in MitoPark mice precipitates most of PD‐like sleep/wake alterations and further confirms the potential role of DA neurotransmission in modulating qualitative and quantitative aspects of sleep/wake behavior.

### 2.2 Hypersomnia in Mitopark Mice Is Not Mediated by Increased Sleep Pressure nor by Altered Homeostatic Regulation of Sleep

**Figure 2:**
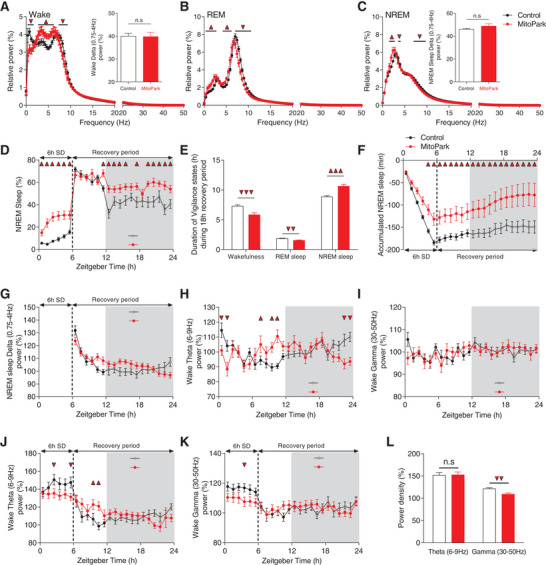
Hypersomnia in MitoPark is not induced by an intrinsic increase in, nor by an altered homeostatic regulation of, sleep need. Power spectral density analysis of A) wake, B) REM, and C) NREM sleep during LD cycle in control (*n* = 19, 10 males) and MitoPark (*n* = 19, 12 males) mice (Two‐way ANOVA revealed group × frequency interaction. *F*_1,128_ (wake) = 4.515, *p* < 0.001; *F*_1,128_ (REM) = 6.919, *p* < 0.001; *F*_1,128_ (NREM) = 3.802, *p* < 0.001; Sidak's post‐hoc analysis, Triangle, *p* < 0.05). Inserts in (A) and (C) show no significant difference in delta power between MitoPark mice and their littermate controls in both A) wake and C) NREM sleep. D) Percentage of time spent in NREM sleep during SD day in MitoPark and littermate mice (*n* = 19 each, Two‐way ANOVA revealed a significant groups × time interaction. *F*_1,23_ = 3.066, *p* < 0.001, Sidak's post‐hoc analysis, *p* < 0.05). E) Total time spent in wake, REM, and NREM sleep during recovery period in MitoPark and control littermates mice (one‐way ANOVA revealed significant decrease of both wake and REM sleep and an increase of NREM sleep in MitoPark mice, *F*_1,49_ (wake) = 13.23, *p* < 0.001; *F*_1,49_ (REM) = 7.84, *p* = 0.007; *F*_1,49_ (NREM) = 21.473, *p* < 0.001). F) Cumulative recovery NREM sleep during SD day (Two‐way ANOVA revealed no significant groups × time interaction, *F*_1,23_ = 0.754, *p* = 0.79). G) Evolution of NREM sleep delta power after 6 h SD expressed as percentage of the mean NREM sleep delta power (0.75–4 Hz) during baseline day (Two‐way ANOVA revealed no significant groups × time interaction, *F*_1,23_ = 1.465, *p* = 0.073). H) Evolution of theta (6–9 Hz) power density during wakefulness in LD in MitoPark and control littermate mice (Two‐way ANOVA revealed a significant groups × time interaction, *F*_1,23_ = 3.66, *p* < 0.001). I) Evolution of gamma (30–50 Hz) power density during wakefulness in LD (Two‐way ANOVA revealed no groups × time interaction, *F*_1,23_ = 0.874, *p* = 0.635). J) Evolution of wake theta (6–9 Hz) power density during SD day (two‐way ANOVA revealed a significant groups × time interaction, *F*_1,23_ = 2.104, *p* = 0.002). K) Evolution of wake gamma (30–50 Hz) power density during SD day (Two‐way ANOVA revealed no significant groups × time interaction, *F*_1,23_ = 1.23, *p* = 0.208). L) Mean power density of theta and gamma during SD (One‐way ANOVA revealed significant decrease of EEG gamma power during SD in MitoPark mice relative to controls. *F*_1,49_ (theta) = 0.006, *p* = 0.94; *F*_1,49_ (gamma) = 9.203, *p* = 0.004). Sidak's post‐hoc analysis, Triangle, *p* < 0.05. Data represent mean ± sem.

What is the neural mechanism by which DA loss induces hypersomnia in MitoPark mice? According to the classical two‐process model of sleep regulation, the amount of time spent in NREM sleep is determined by a homeostatically regulated sleep need which increases during wakefulness and dissipates during sleep.^[^ ^19^, ^20^ ^]^ Slow‐wave Activity (SWA) defined as the spectral power density of EEG delta waves (0.75–4 Hz) during NREM sleep is the best index of sleep depth, while the same marker during wakefulness is the best index of sleep pressure.^[^ ^19^, ^20^ ^]^ We therefore compared the EEG power spectrum of MitoPark and control mice separately for all vigilance states (**Figure** **2**A–C). During wakefulness, MitoPark mice exhibited decreased EEG spectral power at 0.4–1.5 Hz and at 7.4–9.4 Hz and increased power at 2.7–6.25 Hz (Figure 2A). During REM sleep, MitoPark mice showed increased power at 0.4–2.7 Hz and 4.3–6.25 Hz and decreased power density at 7–10.5 Hz (Figure 2B). During NREM sleep, MitoPark mice had increased power density at 1.2–2.7 Hz and decreased power at both 3.5–3.9 Hz and 7–9.8 Hz (Figure 2C). Similar qualitative changes in the EEG spectra were observed during both light and dark phases of the LD cycle (Figure S4, Supporting Information). Interestingly, SWA during both wake and NREM sleep was not significantly increased in MitoPark mice (Figure 2A,C inserts) suggesting that hypersomnia in MitoPark mice is not driven by an intrinsic increase in sleep pressure. To assess the homeostatic modulation of sleep following DA loss, we challenged MitoPark and control littermate mice with a 6 h sleep deprivation (SD) starting from the onset of the light phase. During SD, and despite similar methodology of SD between the two groups (see Experimental Section), MitoPark mice lost significantly less NREM sleep (Figure 2D). After SD, both groups responded by sleeping more during the recovery period relative to baseline. During the first 5 h of the recovery period, no difference was found between MitoPark mice and their littermate controls suggesting an unimpaired homeostatic response. After 5 h however, MitoPark mice spent significantly more time asleep relative to controls (Figure 2D) leading to an overall increase in NREM sleep over the recovery period (Figure 2E). The percentage of REM sleep during the same period was significantly decreased in MitoPark mice (Figure 2E). The increased amount of sleep following SD in MitoPark mice could reflect either an exaggerated homeostatic response or an intrinsic hypersomnia induced by loss of DA neurons. To distinguish between these two possibilities, we compared the accumulated NREM sleep over the SD day in MitoPark and littermate control mice relative to their respective NREM sleep profiles during baseline (Figure 2F). Although both groups had quantitatively different accumulated NREM sleep all over the recovery period as a consequence of the different amount of sleep lost during SD (Figure 2F), the rate of recovery was not significantly affected (Controls; 1.11 ± 0.37% h^−1^. MitoPark; 1.76 ± 0.75% h^−1^; *t*‐test; *t*_48_ = −0.791, *p* = 0.433) suggesting a normal homeostatic response to sleep loss.

Surprisingly, NREM sleep delta power during recovery period was not increased in MitoPark mice relative to controls (Figure 2G). The dynamic of SWA was not significantly different between the two groups all over the recovery period (Figure 2G). These results demonstrate that the hypersomnia in MitoPark mice does not result from an impaired homeostatic process of sleep regulation nor from an intrinsic increase in sleep pressure.

To further confirm this conclusion, we next examined whether MitoPark mice show altered wake‐promoting responses to caffeine and Modafinil (Figure S5, Supporting Information). Caffeine promotes wakefulness by antagonizing the action of adenosine which is considered to be one of the major mediators of homeostatic regulation of sleep in the brain^[^ ^34^ ^]^ while modafinil increases wakefulness through the interaction with several neurotransmitters^[^ ^35^ ^]^ which might be affected in MitoPark mice.^[^ ^36^ ^]^ If MitoPark mice have a normal homeostatic process of sleep regulation, we hypothesize that their response to caffeine will be normal while their response to modafinil might be altered. MitoPark and littermate control mice were administered either 15 mg kg^−1^ of caffeine or saline at the beginning of the light phase (Figure S5A, Supporting Information). As expected, caffeine induced a similar wake response in MitoPark and their littermate controls (Figure S5B,C, Supporting Information). The total amount of wakefulness induced by caffeine during the first 4 h of light phase was not different between the two groups (Figure S5D, Supporting Information). Qualitatively, however, the wake induced in MitoPark mice was different as shown by the spectral power composition of the EEG signal (Figure S5E, Supporting Information). MitoPark mice had reduced power between 0 and 7 Hz (Figure S5E, Supporting Information). The spectral composition of EEG during subsequent NREM sleep was not altered in MitoPark mice (Figure S5F, Supporting Information). These results confirm the unaltered homeostatic regulation of sleep following sleep loss induced by caffeine in MitoPark mice. Contrary to the effect of caffeine, a modafinil challenge generated a different profile in MitoPark mice relative to controls (Figure S5G–I, Supporting Information). Wake induction following modafinil i.p. injection (45 mg kg^−1^) was more powerful and prolonged in MitoPark mice (Figure S5I, Supporting Information) relative to controls (Figure S5H, Supporting Information) leading to a significantly higher amount of wake throughout the entire light phase (Figure S5J, Supporting Information). The spectral power composition of both wake (Figure S5K, Supporting Information) and NREM sleep (Figure S5L, Supporting Information) was also altered in MitoPark mice relative to controls. Taken together, our results show that MitoPark mice do not suffer from a pathological increase in intrinsic sleep pressure nor from an altered homeostatic regulation of sleep following sleep loss. This however does not preclude altered responses to other pharmacologically induced sleep deprivations (i.e., by Modafinil).

### 2.3 Mitopark Mice Have an Impaired Instrumental Wakefulness, and a Lower Threshold of Sleep Induction in Face of Salient Stimuli

For several decades, DA has been linked with the encoding and modulation of motivational drive.^[^ ^27^ ^]^ Recent reports have refined our understanding by showing the role of DA in computing the value of work.^[^ ^27^, ^28^, ^29^, ^30^, ^31^, ^32^ ^]^ According to these studies, DA neurotransmission encodes instrumental wakefulness. That is the invigoration of behavioral response as the animal approaches an intended rewarded target.^[^ ^29^ ^]^ Recently, an electrophysiological EEG marker for such motivated behaviors in mice at the theta and gamma range was characterized.^[^ ^37^ ^]^ We therefore started by computing the dynamic of theta (6–9 Hz) and gamma (30–50 Hz) power density over LD and SD days (Figure 2H–K). Under LD cycle, and consistent with the potential of theta power density to track sleep need during wake,^[^ ^38^ ^]^ control mice displayed a 24 h pattern of theta power with typical decreasing and increasing trends, during respectively, light and dark phases (Figure 2H). MitoPark mice failed to show such pattern leading to significant differences with control mice during the first 2 h of the light phase and the end of both light and dark phases (Figure 2H). The pattern of gamma power was not different in MitoPark mice during LD condition (Figure 2I). During SD however, both control and MitoPark mice increased the percentage of both theta (Figure 2J) and gamma power (Figure 2K). The magnitude of the increase was, however, significantly lower for gamma power in MitoPark mice relative to controls (Figure 2J–L). During the recovery period, wake EEG in MitoPark mice still displayed occasionally altered theta without a significant alteration in gamma power densities (Figure 2J–K). These results suggest indeed that motivated behavior in MitoPark mice might be impaired as a consequence of an inability to mobilize theta and gamma‐rich instrumental wake states.

**Figure 3:**
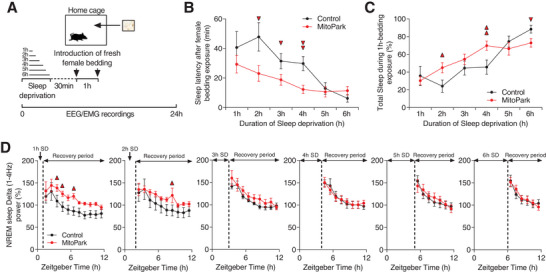
MitoPark mice show impaired arousal responses to salient stimuli. A) Diagram depicting the experimental protocol. B) Sleep latencies after 1 to 6 h SD and following exposure to fresh female bedding in MitoPark (*n* = 11, all males) and control littermate mice (*n* = 8, all males) (unpaired *t*‐test, ▲ *p* < 0.05, ▲▲ *p* < 0,01). C) Total amount of sleep during the 1 h exposure to female bedding after 1 to 6 h SD (unpaired *t*‐test, ▲ *p* < 0.05, ▲▲ *p* < 0,01). D) Delta power during NREM sleep after 1 to 6 h SD (Two‐way ANOVA revealed no groups × time interaction, *F*_1,10_ (1 h SD) = 0.223, *p* = 0.994; *F*_1,9_ (2 h SD) = 0.299, *p* = 0.974; *F*_1,8_ (3 h SD) = 0.211, *p* = 0.989; *F*_1,7_ (4 h SD) = 0.142, *p* = 0.995; *F*_1,6_ (5 h SD) = 0.237, *p* = 0.963; *F*_1,5_ (6 h SD) = 0.27, *p* = 0.929). Data represent mean ± sem.

Based on these results, we hypothesize that the hypersomnia in MitoPark mice might result from impaired DA‐mediated arousal response as a consequence of the degeneration of DA neurons. To test this hypothesis, we subjected MitoPark and littermate control mice to increasing durations of SD (1 to 6 h, starting from ZT0) in order to generate a gradient of wakefulness duration with increasing sleep pressure. 30 min following the end of SD, we introduced fresh salient female bedding into animals’ home cage and determined sleep latency (**Figure** **3**A). The degree of arousal is inversely correlated with the probability of falling asleep and correlates positively with sleep latency. In addition, the strength of instrumental arousal is controlled independently of sleep need^[^ ^39^ ^]^ and reflects the cumulative impact of active wake‐promoting neurons.^[^ ^40^ ^]^ Impaired DA‐mediated instrumental arousal could therefore be reflected as short sleep latencies in face of salient stimuli. As expected, SD shortened the latencies to initiate sleep behavior in both MitoPark and littermate controls (Figure 3B). No significant difference in sleep latencies between the two groups after 1 h SD (Figure 3A). Starting from 2 h SD, however, MitoPark mice had shorter latencies to fall asleep after introducing female bedding (Figure 3B). These latencies were ≈50% shorter relative to the latencies in littermate controls (Figure 3B). After 5 and 6 h SD, both MitoPark and control mice displayed very short sleep latencies with no significant differences between the two (Figure 3B), owing possibly to a ceiling effect. The total amount of sleep (NREM + REM sleep) within the hour of exposure to female bedding was also significantly higher in MitoPark mice following 2 and 4 h SD with no significant difference after 1, 3, and 5 h SD relative to littermate mice (Figure 3C). After 6 h SD, MitoPark mice showed a slight decrease in total sleep relative to control mice. However, both groups showed high percentages (>60%) of sleep indicating again a ceiling effect (Figure 3C). These results confirm our hypothesis and show that MitoPark mice have an impaired capacity to invigorate instrumental arousal and consequently gave a lower threshold of sleep initiation even facing salient stimuli.

Next, we examined whether these different sleep responses between MitoPark and littermate mice are mediated by differential sleep pressure between the two groups during the exposure to female bedding. To do this, we computed NREM sleep delta power in MitoPark and control littermates following SD experiments (Figure 3D). Consistent with our previous data (Figure 2), we found no significant difference in the dynamic of delta (1–4 Hz) power between the two groups after 2–6 h SD (Figure 3D). Only after 1 h SD, during which latencies to initiate sleep were not impaired, does MitoPark show a slight increase in delta power (Figure 3D). These results demonstrate that an increased sleep pressure is not the pathophysiological mechanism behind the low threshold of sleep initiation in MitoPark mice. Collectively, our data demonstrate that loss of DA neurons in MitoPark mice impairs instrumental wakefulness and lowers the threshold of sleep initiation independently of homeostatically regulated sleep need.

### 2.4 The Endogenous Circadian Clock Is Not Functionally Impaired in Mitopark Mice

**Figure 4:**
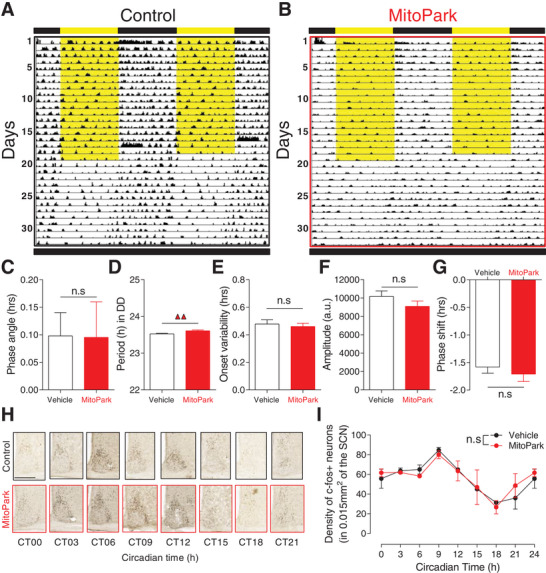
The endogenous function of the circadian clock is not impaired in MitoPark mice. Representative double‐plotted actograms of rest/activity cycles from a A) control littermate and B) MitoPark mouse under LD and DD conditions. C) The phase angle of entrainment in LD was not affected in MitoPark mice (Control, *n* = 24 (12 males); MitoPark, *n* = 22 (10 males); *t*‐test, *t*_44_ = −0.141, *p* = 0.888). D) The endogenous free‐running period of rest/activity rhythm in DD was slightly increased in MitoPark mice (*t*‐test, *t*_44_ = −2.996, *p* = 0.004). E) The onset variability was not affected under DD in MitoPark mice (*t*‐test, *t*_44_ = 0.316, *p* = 0.754). F) The amplitude of rest/activity rhythms in DD was not affected in MitoPark mice (*t*‐test, *t*_44_ = 1.088, *p* = 0.282). G) The phase delay induced by 1 h light pulse at ZT14 was comparable between MitoPark and control littermates (*t*‐test, *t*_44_ = 0.612, *p* = 0.543). H,I) The circadian pattern of c‐fos expression in the suprachiasmatic nucleus, as revealed by immunohistochemistry, is not affected in MitoPark mice (*n* = 3–4 per time point, Two‐way ANOVA, *F*_1,7_ = 0.37, *p* = 0.913). Scale bar in (H) = 250 µm. Data represent mean ± sem.

In addition to orchestrating the timing of sleep/wake behavior, recent studies have implicated the circadian clock in modulating quantitative aspects of sleep/wake cycle.^[^ ^41^, ^42^ ^]^ We therefore examined the properties of the circadian system in MitoPark mice by continuously monitoring rest/activity cycles under both 12 h/12 h LD cycles and constant darkness (**Figure** **4**). Under LD, MitoPark mice showed normal entrainment with no significant difference in phase angle of entrainment compared to control littermates (Figure 4A–C). In DD, and consistent with the modulatory role of midbrain DA on the velocity of the circadian clock,^[^ ^43^ ^]^ MitoPark mice showed a slight but significant elongation of the period of the free‐running rest/activity rhythm (Figure 4D), but both the onset variability (Figure 4E) and the amplitude of the rhythm (Figure 4F) were not affected in MitoPark mice. We also examined photoentrainment by exposing animals to 1 h light pulse at ZT14 before releasing them into constant darkness. No difference was found in the phase delay induced by the light pulse between MitoPark mice and their control littermates (Figure 4G). These results demonstrate that the fundamental functional properties of the circadian clock are not impaired in MitoPark mice. By sampling brain tissue every 3 h over a circadian day, we also revealed a normal circadian pattern of both c‐FOS (Figure 4H,I) and BMAL1 (Figure S6, Supporting Information) expression in the suprachiasmatic nucleus (SCN) of MitoPark mice compared to controls. Collectively, these findings confirm the functional integrity of the circadian clock in MitoPark mice which implies therefore that an impaired clock is unlikely to account for the pathological sleep phenotype in MitoPark mice.

### 2.5 The Direct and Acute Modulation of Sleep/Wake Cycle by Light and Dark Is Not Impaired in Mitopark Mice

**Figure 5:**
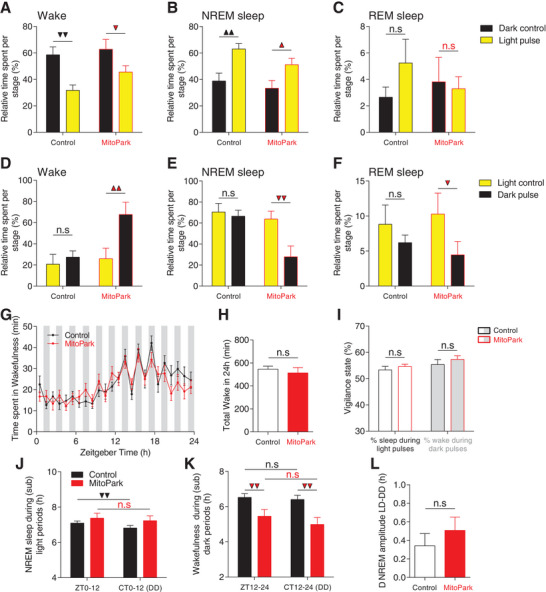
Masking effects of light and darkness on sleep/wake behavior are broadly intact in MitoPark mice. A–C) Quantification of light pulse effects on different vigilance states at ZT14‐15 (control, *n* = 8, MitoPark mice, *n* = 5; *F*_1,1_ (wake) = 0.705; *F*_1,1_ (NREM) = 0.343; *F*_1,1_ (REM) = 1.1; Sidak's post hoc analysis,1 triangle, *p* < 0.05; 2 triangles, *p* < 0,01). D–F) Quantification of dark pulse effects of different vigilance states at ZT2‐3 (*F*_1,1_ (wake) = 3.608; *F*_1,1_ (NREM) = 3.863; *F*_1,1_ (REM) = 0.467. Sidak's post hoc analysis,1 triangle, *p* < 0.05; 2 triangles, *p* < 0,01). G) Time spent in wake state during 1 h light/1 h dark protocol in MitoPark (*n* = 23, 12 males) and control littermate mice (*n* = 13, 8 males) (Two‐way ANOVA revealed no group × time interaction, *F*_1,23_ = 0.956, *p* = 0.522). H) No difference was found in the total amount of wake over 24 h of the 1 h/1 h LD protocol between MitoPark and littermate control mice (unpaired *t*‐test, *p* = 0.306). I) The relative distribution of sleep and wake during light and dark pulses was not different between MitoPark and control littermate mice (unpaired *t*‐test, *p*(%sleep) = 0.207, *p*(%wake) = 0.209). J) The amount of NREM sleep during the subjective light period under DD is decreased relative to the amount during light period under LD12:12 in littermate control mice but not in MitoPark mice (One‐way RM ANOVA, *F*_1,25_ (control) = 11.568, *p* = 0.002; *F*_1,25_ (MitoPark) = 2.295, *p* = 0.143). K) The amount of wake during the subjective dark period under DD is comparable to the amount during dark period under LD12:12 in both control and MitoPark mice (One‐way RM ANOVA, *F*_1,25_ (control) = 0.819, *p* = 0.374; *F*_1,25_ (MitoPark) = 2.319, *p* = 0.141). L) The differences in amplitudes of sleep/wake cycle under LD and DD is not significantly different between MitoPark mice and their control littermates (unpaired *t*‐test, *p* = 0.203). Data represent mean ± sem.

Alongside its entrainment effects on sleep/wake cycle, light and darkness modulate acutely the quality and quantity of vigilance states through a mechanism known as masking.^[^ ^22^, ^23^, ^24^ ^]^ In nocturnal mice, darkness is wake promoting while light strongly induces sleep.^[^ ^44^ ^]^ A possible mechanism therefore behind hypersomnia in MitoPark mice could be an impaired masking effect of light and dark. To investigate this possibility, we examined sleep induction in MitoPark and littermate controls by exposing them to a 1 h light pulse 2 h after “light‐off” (ZT14). The distribution of different vigilance states during the 1 h light exposure was comparable between MitoPark and control littermates (**Figure** **5**A–C). In both groups, light exposure significantly decreased wake (Figure 5A) and increased NREM sleep (Figure 5B) without significantly affecting REM sleep percentages (Figure 5C). The fact that hypersomnia in MitoPark mice was mainly restricted to the dark phase (Figure 1 and Figure S1, Supporting Information) might suggest an impaired ability of darkness to awake mice. To explore this possibility, we exposed MitoPark and control mice to a 1 h dark pulse 2 h after “light‐on” at ZT02. Interestingly, the phenotype obtained is the opposite of what we expect if this mechanism accounts for the hypersomnia in MitoPark mice (Figure 5D–F). The 1 h dark pulse significantly increased wake (Figure 5D) and decreased both NREM sleep (Figure 5E) and REM sleep (Figure 5F) in the MitoPark mice. In control mice, no significant effects of the dark pulse on vigilance states were observed (Figure 5D‐F). To probe in more detail the effects of light and dark on sleep/wake cycle across the whole 24 h day, we exposed MitoPark and control mice to ultradian 1 h light/1 h dark cycles over 24 h (Figure 5G). Under such light regime, mice are unable to entrain to the short 1 h/1 h LD cycle allowing therefore light and dark pulses to fall within all phases of the circadian cycle.^[^ ^24^ ^]^ In both MitoPark and control littermate mice, alternating light and dark pulses were equally efficient in modulating the amount of vigilance states (Figure 5G) with no significant impact on the total amount of wake over the 24 h period (Figure 5H), nor on the relative distribution of wake and sleep states percentages throughout all light and dark pulses (Figure 5I). These results demonstrate that the acute modulation of sleep/wake by light and darkness is not affected in MitoPark mice.

Recently, Hubbard et al. showed that masking effects of light and dark on sleep/wake cycles are not only acute but sustained over time and account for half of the overall changes to the amplitude of sleep/wake rhythms.^[^ ^45^ ^]^ To probe this chronic masking effects of LD pulses, we compared the distribution of NREM sleep during light phase of LD cycle to its corresponding subjective day under DD condition. As expected, in control mice, a significant decrease in total NREM sleep was shown during the subjective day in DD compared to the light phase of the LD cycle (Figure 5J). MitoPark mice failed to show such difference owing probably to the already saturated amount of NREM sleep in MitoPark mice during these phases. The comparison of the amounts of wake during the dark phase of LD cycle to the subjective night in DD showed indeed a significant decrease under DD in MitoPark mice (Figure 5K) with no significant difference in NREM amplitude difference between LD and DD in MitoPark mice relative to controls (Figure 5L). Taken together, these data show that the masking effects of light and darkness on the sleep/wake cycle are broadly intact in MitoPark mice and could therefore not account for their hypersomnia phenotype.

### 2.6 Modulation of NREM Sleep by Behavioral Valence Is Impaired in Mitopark Mice and Is Uncoupled from the Homeostatic Process of Sleep/Wake Regulation

**Figure 6:**
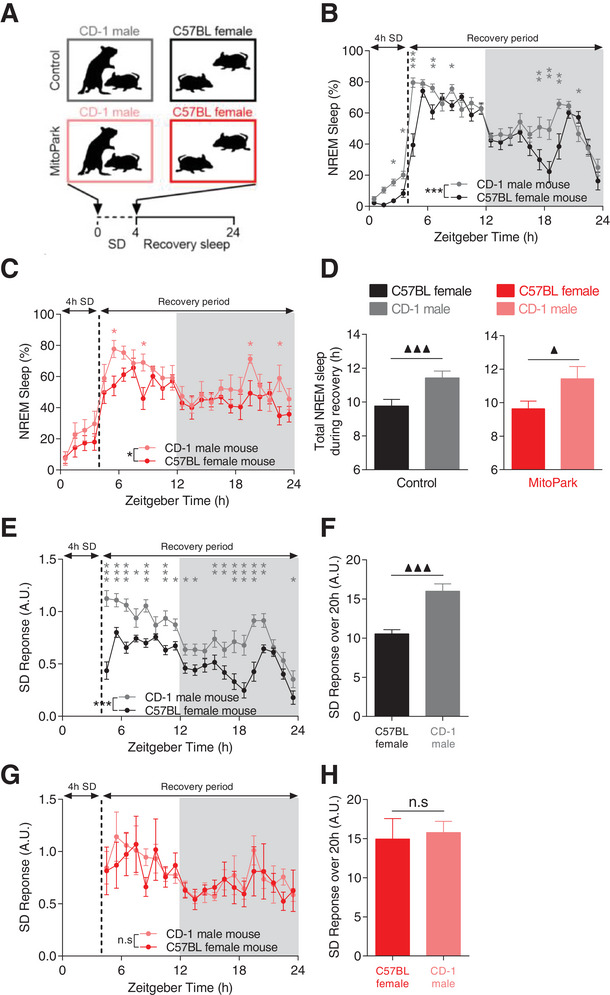
The modulation of sleep/wake behavior by motivational valence is impaired in MitoPark mice. A) Schematic diagram depicting the experimental protocol. Percentage of time spent in NREM sleep during and after SD through the interaction with either CD‐1 male or conspecific female B) in control (*n* = 17, all males) and C) MitoPark mice (*n* = 8, all males) (two‐way RM ANOVA revealed significant groups × time interaction for control (*F*_1,23_ = 4.77, *p* < 0.001) and not for MitoPark mice (*F*_1,23_ = 0.784, *p* = 0.746). D) Total amount of NREM sleep during recovery period in control and MitoPark mice following SD (One‐way RM ANOVA revealed significant difference in both control (*F*_1,16_ = 42.38, *p* < 0.001) and MitoPark mice (*F*_1,7_ = 8.992, *p* = 0.024). E) Hourly and F) overall normalized SD response calculated as [recovery sleep/lost sleep during SD] in control littermate mice (in (E): two‐way RM ANOVA, *F*_1,19_ = 4.673, *p* < 0.001; in (F): one‐way RM ANOVA, *F*_1,16_ = 31.878, *p* < 0.001). G,H) Same as in (E,F) but for MitoPark mice (in (G): two‐way ANOVA, *F*_1,19_ = 0.438, *p* = 0.979; in (H): one‐way RM ANOVA, *F*_1,7_ = 1.612, *p* = 0.251). Data represent mean ± sem.

Recent studies conducted in several species have described remarkable modulation of sleep quantity by contextual motivation and valence.^[^ ^46^, ^47^, ^48^, ^49^, ^50^, ^51^, ^52^, ^53^, ^54^, ^55^, ^56^ ^]^ Given that the DA system encodes, in addition to contextual salience, the value and valence of behavior,^[^ ^27^, ^28^, ^29^, ^30^, ^31^, ^32^ ^]^ we asked whether the modulation of sleep/wake cycle by motivational valence is impaired in MitoPark mice. To probe this question, singly‐housed MitoPark and control littermate mice were challenged with two SD protocols with opposite valences for 4 h staring from the beginning of the light phase of LD cycle (**Figure** **6**A). The SD protocol with positive valence consisted of introducing a conspecific female on multiple occasions into the animals’ cage for 4 h while the SD with negative valence consisted of an acute social defeat induced by introducing an aggressive CD‐1 male mouse (see Experimental Section for details). In controls and, to a lesser extent in MitoPark mice, the two SD protocols lead to different sleep rebound phenotypes (Figure 6B,C). After SD, mice that underwent social defeat with CD‐1 male spent significantly more time in NREM sleep at multiple instances during recovery period relative to mice that were sleep deprived with female interaction (Figure 6B,C). This led to a significantly higher cumulative amount of NREM sleep (by ≈ 2 h) during recovery period in CD‐1 male‐exposed relative to female‐exposed mice (Figure 6D). Corroborating previous studies,^[^ ^57^ ^]^ SD with CD‐1 male suppressed significantly REM sleep during the first 6–7 h following SD in both control and MitoPark mice (Figure S7, Supporting Information). These results reveal the powerful impact that the valence of behavior during wakefulness has on the quality and quantity of subsequent sleep.

In control mice, CD‐1 male‐exposed mice slept significantly more during recovery despite having lost less sleep during SD relative to female‐exposed mice (Figure 6B,C). These results challenge the classical homeostatic model of sleep regulation that considers time spent awake as the main determinant of sleep pressure.^[^ ^19^, ^20^ ^]^ In MitoPark mice, no significant difference in the remaining amount of NREM sleep during SD (Figure 6C). To account for this, we normalized the percentage of NREM sleep during recovery period relative to the percentage of NREM sleep lost during SD (see Experimental Section). In control mice, the two SD protocols led to contrasting SD responses with significantly higher responses in CD‐1 male‐exposed relative to female‐exposed mice throughout the recovery period (Figure 6E). The total SD response during recovery was consequently significantly higher in CD‐1 male‐exposed compared to female‐exposed mice (Figure 6F). In MitoPark mice, this differential modulation of SD response by behavioral valence was absent (Figure 6G) with no significant difference in the overall SD response during recovery between CD‐1 male‐ and female‐exposed MitoPark mice (Figure 6H). These results demonstrate the primordial role of an intact DA signaling in mediating sleep modulation by motivational valence.

**Figure 7:**
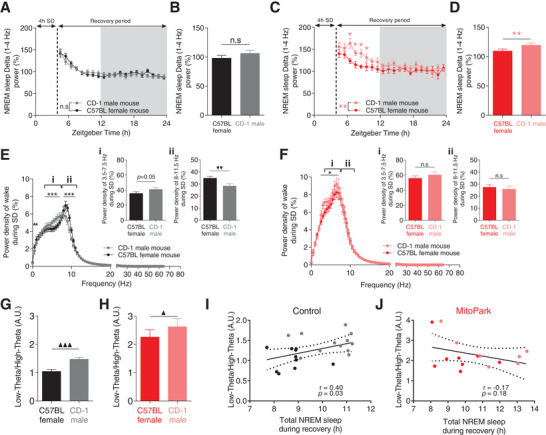
The modulation of NREM sleep by behavioral valence is significantly correlated with cortical EEG theta power. A,B) Surprisingly, CD‐1 exposed versus female‐exposed control littermate mice showed similar dynamic of NREM sleep delta power during recovery period despite displaying different sleep rebound phenotypes following SD (*n* = 17, all males, in (A): two‐way RM ANOVA revealed no significant difference. *F*_1,19_ = 1.973, *p* = 0.573; in (B): one‐way RM ANOVA, *F*_1,16_ = 3.507, *p* = 0.081). C,D) Same as in (A,B) but for MitoPark mice (*n* = 8, all males, in (C): two‐way RM ANOVA revealed significant difference *F*_1,19_ = 1.72, *p* = 0.005; in (B): one‐way RM ANOVA, *F*_1,7_ = 12.665, *p* = 0.012). E) Power spectral density of EEG during SD with CD‐1 male and conspecific female in control littermate mice (two‐way RM ANOVA revealed significant groups × frequency interaction, *F*_1,128_ = 5.446, *p* < 0.001, Sidak's post‐hoc analysis, ***p < 0.001. Relative to conspecific female‐exposed mice, CD‐1 male‐exposed control mice showed Ei) higher and Eii) significantly lower power densities of respectively low theta (3.5–7.5 Hz) and high theta (8–11.5 Hz) (One‐way RM ANOVA, *F*_1,10_ (low theta) = 4.949, *p* = 0.05. *F*_1,10_ (high theta) = 17.102, *p* = 0.002). F) Same as (E) but for MitoPark mice (F: two‐way RM ANOVA, *F*_1,128_ = 2.203, *p* < 0.001. Fi,ii: one‐way RM ANOVA, *F*_1,7_(low theta) = 2.955, *p* = 0.136, *F*_1,7_ (high theta) = 0.667, *p* = 0.445). G,H) [Low theta/high theta] significantly and powerfully discriminated between CD‐1 male exposed and female‐exposed littermate G) control mice H) but only slightly in MitoPark mice (one‐way RM ANOVA, *F*_1,10_ (control) = 33.333, *p* < 0.001, *F*_1,7_ (MitoPark) = 8.097, *p* = 0.029). Correlations between [low theta/high theta] ratios and total NREM sleep during recovery period in I) control and J) MitoPark mice (Spearman correlation). Data represent mean ± sem.

Wake experience and intensity are known to modulate sleep need^[^ ^39^, ^57^ ^]^ and social stress like the one we used in CD‐1 male‐exposed mice increases sleep amount.^[^ ^58^, ^59^ ^]^ Based on the homeostatic process of sleep regulation, we expected CD‐1 male exposed mice to have higher sleep pressure relative to conspecific female‐exposed mice. Surprisingly, however, this was not the case (**Figure** **7**A–D). In control mice, no significant difference in NREM sleep delta (1–4 Hz) power was observed throughout the recovery period in CD‐1 male relative to female‐exposed mice (Figure 7A,B). In MitoPark mice however, a significant increase was found in both the hourly (Figure 7C) and the overall (Figure 7D) NREM sleep delta power during recovery period. No significant correlation was found between NREM sleep delta power and the total amount of NREM sleep during recovery in both littermate controls (Figure 4A) and MitoPark mice (Figure 4B). These results demonstrate that in healthy control mice, the modulation of sleep rebound by behavioral valence is uncoupled from SWA modulation. In MitoPark mice, sleep modulation by motivational valence is lost without affecting the homeostatic regulation of sleep.

### 2.7 EEG Spectral Characteristics during Wakefulness Reliably Discriminate between Contrasting Behavioral Valences and Are Sensitive to Dopaminergic Signaling

EEG is a powerful tool to discriminate between a wide spectrum of arousal levels, sensorimotor processing modalities, and behavioral states associated with different wake states.^[^ ^60^ ^]^ Because cognitive processing of contextual valence occurs during wakefulness, we analyzed and compared spectral properties of wake EEG in CD‐1 male versus female‐exposed mice during SD. Interestingly, distinct profiles of EEG spectral power were found for each SD protocol in littermate control mice (Figure 7E). More specifically, there was a differential modulation of low (3.5–7.5 Hz) and high theta (8–11.5 Hz) power densities by behavioral valence (Figure 7E). CD‐1 male exposed mice had an EEG with higher power density of low theta (Figure 7Ei) and significantly lower power density of high theta (Figure 7Eii) relative to female‐exposed mice. In MitoPark mice, this differential modulation of EEG theta power was severely affected (Figure 7F) with no significant difference in both low theta power (Figure 7Fi) and high theta power (Figure 7Fii) between CD‐1 male versus female‐exposed MitoPark mice. In control mice, low theta power density was not significantly correlated with total NREM sleep during recovery period (Figure S8C, Supporting Information). However, a significant negative correlation was found between high theta power density and total NREM sleep during recovery period (Figure S8E, Supporting Information). Both correlations are not significant in MitoPark mice (Figure S8D,F, Supporting Information). Taken together, these results demonstrate that motivational valence during sleep deprivation specifically modulates cortical theta rhythms and that this modulation—mainly at the high theta range—reliably predicts the amount of subsequent NREM sleep rebound in healthy control animals. Furthermore, results from MitoPark mice highlight the role of DA neurotransmission in mediating behavioral valence‐related modulation of both sleep and cortical theta oscillations.

Because SWA did not reliably discriminate between sleep rebound phenotypes of the two SD protocols in control mice (Figure 7A,B). We sought to characterize an alternative EEG marker using the differential modulation of low and high theta power we found between the two SD protocols (Figure 7E,F). We computed ratios of power densities of [low theta/high theta] and compared them across the two SD protocols in both control and MitoPark mice (Figure 7G,H). In control mice, a significantly high [low theta/high theta] ratio was found during SD with CD‐1 male mice compared to SD with conspecific female mice (Figure 7G). In MitoPark mice, the difference in this ratio between the two SD protocols was reduced (Figure 7H). [low theta/high theta] correlated positively with the total amount of NREM sleep rebound after the two SD protocols in control littermate mice (Figure 7I). In MitoPark mice, this correlation was flatter and not significant (Figure 7J). These results demonstrate that the ratio of the power density of [low theta (3.5–7.5 Hz)/high theta (8–11.5 Hz)] during SD is a reliable EEG marker for predicting the modulation of subsequent NREM sleep rebound by behavioral valence. Additionally, the reliability of this EEG marker is contingent on an intact and functional DA signaling.

## 3 Discussion

As a neuromodulator, DA is strongly involved in the regulation of a variety of functions ranging from sensorimotor functions, emotional regulation to more integrative cognitive processes such as memory and attention.^[^ ^61^, ^62^ ^]^ Not surprisingly, therefore, dysfunctional DA neurotransmission is associated with several neurological and neuropsychiatric diseases.^[^ ^63^ ^]^ Virtually all patients suffering from these disorders display debilitating sleep problems leading to a significant decrease in quality of life.^[^ ^1^, ^2^, ^3^, ^4^ ^]^ Efficient mechanism‐based therapies are currently unavailable leading to increased societal burden associated with hospitalization and medical care.^[^ ^64^ ^]^ In PD patients, for example, sleep alterations are predominant and among the most debilitating NMS of the disease.^[^ ^5^, ^6^ ^]^ While it is clear that several factors, including intrinsic neuronal pathology, secondary impact of motor and NMS, and medication, are implicated in the etiology of sleep problems in PD, the relative contribution of DA dysfunction is still not fully appreciated.^[^ ^65^ ^]^

Ever since the discovery of DA as an independent neurotransmitter in the brain,^[^ ^66^, ^67^ ^]^ lesional,^[^ ^8^, ^9^ ^]^ pharmacological^[^ ^8^, ^9^ ^]^ and more specific chemo/optogenetic^[^ ^10^, ^11^, ^12^, ^13^, ^14^, ^15^, ^16^, ^17^ ^]^ preclinical manipulations, that up or down‐regulate DA neurotransmission have revealed the potent role of DA in regulating arousal and motivational wakefulness. In the clinic, however, the consequences of dopaminomimetic drugs on sleep/wake state unravel more complex dose‐ and targeted receptors‐dependent effects.^[^ ^68^ ^]^ Additionally, whether DA effects are mediated through the interaction with the homeostatic or circadian processes of sleep regulation, masking effect of light and dark, motivational arousal, or a combination of more than one or all of these factors, is still unknown.

We have demonstrated here that sleep alterations associated with parkinsonism emerge within a background of unaltered homeostatic and circadian regulation of sleep/wake cycle. In fact, a hypersomniac phenotype in MitoPark mice was associated with normal sleep pressure showing that an intrinsic increase in sleep need is not the pathophysiological mechanism behind hypersomnia in MitoPark mice. Furthermore, the acute effects of light and darkness on sleep/wake architecture showed minimal alterations with broadly normal overall 24 h impact. This also demonstrates that impaired masking of light and dark does not contribute to a pathological sleep phenotype in MitoPark mice. Consistent with the role of DA in modulating motivational drive,^[^ ^27^, ^28^, ^29^, ^30^, ^31^, ^32^ ^]^ MitoPark mice showed an arousal deficit even in a salient environment leading to low thresholds of falling asleep. To our knowledge, this is the first time a systematic investigation of all potential mechanisms of sleep/wake regulation was undertaken in a pathological context of Parkinsonism. Furthermore, our valence modulation experiments allow us to appreciate the powerful impact of contextual valence during sleep deprivation on the quality and quantity of subsequent sleep rebound. This motivational valence‐related modulation of sleep amount was contingent on a functional DA signaling and was uncoupled from homeostatically‐regulated sleep pressure. Finally, we discovered for the first time a reliable wake EEG index at theta range that was also sensitive to DA loss and significantly predicted the modulation of NREM sleep rebound by behavioral valence.

The necessary daily amount of sleep is tightly regulated by the homeostatic process of sleep regulation.^[^ ^19^, ^20^, ^21^, ^69^ ^]^ Although this daily quota of sleep is different across species,^[^ ^70^, ^71^ ^]^ any deviation from obtaining the right amount of sleep is associated with several detrimental health consequences in all tested animals.^[^ ^69^ ^]^ In humans, for example, a recent large longitudinal study has shown a global cognitive decline in individuals with either insufficient (≤4 h per night) or excessive (≥10 h per night) sleep duration.^[^ ^72^ ^]^ Exceptions to this general rule however occur in settings where deviations from the necessary sleep quota are adaptive. Several recent studies both in the wild and in lab conditions have documented remarkable examples of sustained sleep loss without a decrease in neurobehavioral performances and without the typical subsequent sleep rebound.^[^ ^46^, ^47^, ^48^, ^49^, ^50^, ^51^, ^52^, ^53^, ^54^, ^55^, ^56^ ^]^ All these examples involve a clear modulation of motivational drive either artificially (in the lab) or through natural changes in ecological demands. As we all know, motivated human subjects can also stay awake for several days by keeping themselves busy with pleasurable activities (i.e., the famous example of Randy Gardner who stayed continuously awake for 11 days^[^ ^73^ ^]^). The opposite sleep profile happens when the valence of behavior is reversed. Likewise, some studies in rodents have shown exaggerated sleep responses when sleep deprivation is done through stressful means.^[^ ^57^, ^58^, ^59^ ^]^ Our study identifies DA neurotransmission as a potential neural substrate that mediates sleep modulation by motivational valence. Interestingly, recent studies have implicated several nodes of the DA neurocircuitry in the modulation of the sleep/wake cycle. These include dorsal^[^ ^17^, ^74^ ^]^ and ventral striatum,^[^ ^75^ ^]^ VTA,^[^ ^10^ ^]^ PVN,^[^ ^76^ ^]^ RMT,^[^ ^13^ ^]^ VP,^[^ ^77^ ^]^ and the cortex.^[^ ^78^ ^]^ All these centers are also known to encode valence.^[^ ^79^ ^]^ We therefore suggest that neuronal computation and integration of behavioral valence is a central component in modulating the quality and quantity of sleep. Consistent with the role of midbrain DA in modulating mood,^[^ ^80^, ^81^, ^82^ ^]^ neurodegeneration of DA neurons in MitoPark mice induces anxiety and depression.^[^ ^83^ ^]^ The dominance of these depressive moods during wake in MitoPark mice might therefore explain their hypersomnia phenotype. This pathophysiological mechanism could also explain the dramatic decrease of REM sleep in MitoPark mice given the impact of negative valence (i.e., CD‐1 male exposure) on REM sleep (Figure S7, Supporting Information). An alternative, yet not exclusive mechanism for reduced REM sleep in MitoPark mice might be a direct involvement of DA in generating REM sleep. This mechanism is supported by studies showing that midbrain DA neurons are highly active during REM sleep^[^ ^84^, ^85^, ^86^ ^]^ and by a recent study implicating DA selectively in basolateral amygdala in promoting REM sleep.^[^ ^87^ ^]^

SWA and EEG power density of delta waves during wake are the most accurate electrophysiological markers of respectively sleep depth and sleep pressure.^[^ ^19^, ^20^ ^]^ Yet our study revealed a strong modulation of sleep by motivational drive and its hedonic valence that is uncoupled from, and not reflected in, the dynamic of SWA in control animals. Interestingly, such disconnection between SWA and sleep depth or amount has been described in several examples where motivational valence is modulated.^[^ ^46^, ^49^, ^59^, ^88^ ^]^ This suggests that SWA is not a reliable index to track the modulation of sleep by motivational valence. In MitoPark mice however, SWA mirrored the changes in sleep rebound following SD with CD‐1 male and female mice. A plausible explanation for these results is that in control mice, DA is mobilized to cope with the stress induced by the interaction with CD‐1 male. In such conditions, DA might antagonize the buildup of sleep pressure. The loss of this DA‐mediated antagonism of sleep pressure in MitoPark mice will result in an exaggerated accumulation of sleep pressure in stressful and negatively‐charged conditions relative to positively‐charged and enjoyable conditions. This mechanism is consistent with the well‐established role of midbrain DA in mediating adaptive behaviors^[^ ^28^, ^82^, ^89^ ^]^ and in antagonizing the actions of somnogenic factors in the brain such as adenosine.^[^ ^90^ ^]^

The biological meaning of different brain rhythms during wake is still a hotly debated topic in Neuroscience.^[^ ^91^, ^92^ ^]^ The build‐up of SWA in wake EEG has been correlated with attentional lapses and impaired behavioral performances that occur during prolonged wakefulness.^[^ ^93^, ^94^ ^]^ Our study shows that the power density of different theta frequencies during wake is differently modulated by motivational valence and correlates significantly with the amount of subsequent sleep. This demonstrates that these theta bands during motivationally‐charged and sustained wake could reliably inform about the intensity of subsequent sleep. Consistent with this conclusion, other studies have also linked cortical theta with processing of value information.^[^ ^95^ ^]^ Interestingly, power densities of both low (4.5–8.5 Hz) and high theta (8.5–12.5 Hz) increase monotonically in the frontal derivations of human EEG during prolonged wakefulness.^[^ ^96^ ^]^ These studies, however, did not link these EEG theta alterations with mood state changes during SD or with the quantity and quality of subsequent sleep. Future experiments in both humans and animals should shed more light on the specificity of cortical theta rhythms during motivationally charged wake states in encoding sleep modulation by behavioral valence.

Our results hold important translational insights into several neurological and neuropsychiatric disorders with dysfunctional DA neurotransmission. Consistent with our results in MitoPark mice, PD patients experience several circadian alterations that start even before clinical diagnosis. Yet, they do not suffer from an impaired central circadian clock except in later stages of the disease.^[^ ^97^, ^98^ ^]^ Similarly, the overall hypersomnia in PD patients is not the result of an increased homeostatic sleep need since the few studies that explored spectral proprieties of sleep in PD patients showed either a normal^[^ ^99^ ^]^ or even a decrease in SWA during NREM sleep.^[^ ^100^ ^]^ Like PD patients, most seasonal affective disorder patients complain of hypersomnia and daytime somnolence with intact circadian and homeostatic regulation of sleep.^[^ ^101^, ^102^ ^]^ In light of our findings, we propose that DA‐related motivational deficits that are well‐documented in these patients are equally responsible for the abnormal sleep phenotypes in these disorders independently of circadian and homeostatic processes.

MitoPark mice were developed as an attempt to generate a mouse model with parkinsonism closely resembling PD.^[^ ^26^ ^]^ MitoPark mice replicate most of the motor aspects of PD including; the progressive age‐dependent neurodegeneration of DA neurons in both VTA and Substantia nigra (SN, Figure S9, Supporting Information) that parallels the progressive emergence of motor disabilities and the therapeutic responsiveness to L‐DOPA intake.^[^ ^26^ ^]^ A recent study implicated DA neurons within the dorsal raphe nucleus in modulating motivational arousal.^[^ ^12^ ^]^ Downregulation of these DA neurons induces hypersomnia which, unlike in MitoPark mice, was associated with a significant increase in SWA.^[^ ^12^ ^]^ These findings imply that the contribution of these DA neurons to the sleep/wake phenotypes we observed in MitoPark mice is unlikely. This conclusion is further corroborated by the normal histochemical aspect of these neurons in MitoPark mice (Figure S9, Supporting Information). Similar to PD patients, recent studies have also documented several NMS in MitoPark mice.^[^ ^83^ ^]^ In addition to these features, our study reveals a high face validity of MitoPark mice in replicating most sleep alterations experienced by PD patients. The lack of RBD in MitoPark mice is consistent with the non‐DAergic pathophysiological mechanism behind RBD recently shown in several neurocircuit investigations.^[^ ^7^, ^103^, ^104^, ^105^ ^]^ Similarly, nocturnal insomnia has been associated with some NMS and DAergic therapies in PD patients^[^ ^68^ ^]^ which were not investigated in our study. Taken together with published literature, our study positions the MitoPark mouse as a suitable model to investigate therapeutic interventions and to develop efficient treatments against intrinsic sleep alterations in several DA‐related motivational disorders such as PD.

In summary, while it is well‐established that sleep alterations precipitate motivational and mood disturbances,^[^ ^106^ ^]^ our study showed that inversely, contextual valence is a strong factor in modulating sleep/wake behavior. This modulation was uncoupled from the classical circadian and homeostatic processes of sleep/wake regulation and was, at least partly, mediated by midbrain DA neurotransmission and could reliably be tracked by cortical theta power. We also showed that a pathological hypodopaminergic state results in a motivational deficit that accounts for sleep alterations in a well‐established animal model of PD. These results demonstrate that the neural network encoding value combines information about motivational valence in order to shape the quality and quantity of sleep/wake behavior. Future studies should investigate by which mechanisms this valence network senses and/or integrates motivational valence and broadcast this information to the subcortical circuitry responsible for sleep/wake regulation.^[^ ^107^ ^]^

## 4 Experimental Section

### Subjects

Both male and female MitoPark mice aged 12–28 weeks were used in this study. The generation of MitoPark mice was done as previously described.^[^ ^26^, ^108^ ^]^ Briefly, DAT‐Cre mice were crossed with mice carrying Loxp‐flanked Tfam (Tfam Loxp) to generate mice with a homozygous deletion of Tfam selectively in midbrain DA neurons (DAT‐Cre/+, TfamLoxp//TfamLoxp). Littermates of both sexes in which one or both Tfam alleles were Loxp‐flanked (TfamLoxp//TfamLoxp; TfamLoxp//Tfam) served as controls. For genotyping, two PCR reactions were performed. For DAT‐Cre genotype, one forward (F‐5′‐CATGGAATTTCAGGTGCTTGG) and two reverse primers (R1‐5′‐CATGAGGGTGGAGTTGGTCAG and R2‐5′‐CGCGAACATCTTCAGGTTCT) to separate heterozygous DAT‐Cre mice which have two bands at 470 and 310 base pairs from wildtype mice showing only the smaller band. Genotyping for TfamLoxp//Loxp was carried out using one forward (F‐5′‐CTGCCTTCCTCTAGCCCGGG) and two reverse primers (R1‐5′‐GTAACAGCAGACAACTTGTG and R2‐5′‐CTCTGAAGCACATGGTCAAT). 35 cycles were run at 95 °C for 30 s, 55 °C for 30 s, and 72 °C for 45 s. The PCR products were separated using electrophoresis on 1% agarose gels and were visualized with UV after ethidium bromide staining.

Animals were housed on a 12 h/12 h LD cycle (lights on at 6 am) with ad libitum access to food and water. Animal husbandry and experiments were performed in accordance with the animal core committee of both Leiden University Medical Center (approved protocol DEC N‐14220) and the University of Tsukuba (approved protocol ID #180094). Extra effort was made to minimize the number of animals used as well as unnecessary suffering or distress.

### EEG/EMG Recordings

Mice were implanted with chronic EEG and EMG electrodes. EEG screws were inserted through craniotomy holes in frontal (AP, +1.5 mm; ML, +1 mm) and parietal (AP, −3.5 mm; ML, +2.5 mm) regions. Two additional screws were inserted in lateral‐parietal regions and served as implant support. Two insulated, Teflon‐coated silver wires were inserted bilaterally into the trapezius muscles and served as EMG electrodes. The whole implant was fixed to the skull using dental cement. Mice were allowed to recover from surgery on a heating‐pad, then transferred to their residence room for full recovery (at least 10 days) prior to starting behavioral experiments or habituation to EEG/EMG tethers and recording chambers. EEG/EMG recordings started at the beginning of the light phase and lasted for as long as needed depending on the experiment (see below).

### Nest‐Building Experiment

To assess nest‐building behavior in MitoPark mice, the old nest was removed from animal's cage and new nesting material was introduced. Nest building performances were evaluated 4 h later using the five‐point scale developed by Deacon et al., 2006.^[^ ^109^ ^]^ EEG/EMG recordings were recorded continuously during this experiment.

### 6 h Sleep Deprivation

For the evaluation of the homeostatic processes shown in Figure 2, animals were sleep deprived for 6 h starting from light's on using the enrichment of the environment in order to stimulate spontaneous exploratory wake behavior. Mice were monitored online through EEG/EMG signals and video cameras. Whenever mice show signs of entering NREM sleep (i.e., sleep posture and/or noticeable increase in slow wave amplitude), new material (food, clean bedding, water, toys, and tissue) was introduced to the animal's cage. Touching animals was strictly avoided to minimize confounding stress.

### Caffeine and Modafinil Treatments

For the caffeine/modafinil experiments shown in Figure S5, Supporting Information, 24 h after vehicle (Propylene glycol or saline) treatment, MitoPark, and control littermate mice were injected i.p with either caffeine (15 mg kg^−1^, Sigma Aldrich, Cat#014k0036, USA) or modafinil (45 mg kg^−1^, Sigma Aldrich, USA). Caffeine was dissolved in sterile saline (0.9%) while modafinil was dissolved in propylene glycol (Sigma Aldrich, Cat#P4347, USA). EEG/EMG signals were recorded to assess the impact of these drugs on the sleep/wake cycle.

### Determination of Arousal Strength under Different Conditions of Sleep Pressure

To assess arousal responses of mice under different levels of sleep pressure (Figure 3), MitoPark and control mice were challenged to increasing durations of sleep deprivation (1 to 6 h) using the same protocol that was used for 6 h SD. At least 2 days separated successive SD experiments. 30 min after the end of SD, fresh female bedding was introduced to animal's cage for 1 h. Sleep latency and total amount of different vigilance states during this hour were calculated. Sleep latency is defined as the time separating the introduction of female bedding and the appearance of the first 20 s epoch of NREM sleep.^[^ ^39^ ^]^

### Assessment of Behavioral Circadian Rhythms

Circadian rhythms were assessed by monitoring 24 h rest/activity rhythms using previously described methods.^[^ ^108^, ^110^ ^]^ Cages with individually housed animals were equipped with passive infrared motion captors and data were recorded using a Clocklab data acquisition system (Actimetrics) and stored on a computer in 1‐min bins. Food and water were available ad libitum during the experiment. Mice were first exposed to a 12 h light::12 h dark (LD) cycle for 2 weeks to assess rest/activity rhythms and photoentrainment. Animals were then released into constant darkness (DD) for the following 2 weeks in order to assess the intrinsic proprieties of rest/activity rhythms. Subsequently, animals were re‐entrained to a 12 h/12 h LD cycle. To assess circadian entrainment, mice were exposed to 1 h light pulse at ZT14 before they were released to DD.

Clocklab was used for circadian data analyses. Photoentrainment to LD cycle was estimated by computing phase angle of entrainment defined as the mean of the differences between light‐offs and activity onsets. Chi‐squared periodogram analysis^[^ ^111^ ^]^ was used to calculate the endogenous period of rest/activity rhythm under DD conditions. The amplitude of rest/activity rhythms, defined as peak‐to‐nadir difference was extracted from the peak of the chi‐squared periodogram. Finally, to assess re‐entrainment after phase shift, activity onsets were used as a marker of entrainment and correspond to the average clock or circadian time of locomotor activity onset. After each light regime change, the first 1–2 days were excluded for analysis to account for transients.

### Assessment of Masking Effects of Light and Dark on Sleep/Wake Behavior

While animals were monitored in their EEG/EMG chambers, two light regimes were used to assess sensitivity of light and darkness to modulate sleep/wake behavior. The first protocol consisted of exposing mice to 1 h light and 1 h dark pulses at respectively ZT14‐15 and ZT2‐3. Percentages of vigilance states during these pulses were compared to the corresponding time interval on the preceding baseline day for each animal. The second protocol consisted of exposing animals to a 1 h:1 h LD cycle for 24 h. Total amount of sleep and wake states as well as their relative distribution within respectively light and dark phases was analyzed.

### Assessment of Valence‐Related Modulation of Sleep/Wake Behavior

Two protocols of SD were used to expose MitoPark and control mice to opposite valence‐charged wake experiences. Individually‐housed mice were sleep deprived for 4 h starting from light‐on. The negatively‐charged SD protocol consisted of an acute social‐defeat stress using aggressive CD‐1 male mice. CD‐1 mice were generously donated by Tokuda Akihisa. Each mouse was subjected to 4 social‐defeat trials over the 4 h SD. Each trial started at the beginning of each hour of the 4 h SD and lasted 10 min. A CD‐1 male mouse was introduced into the home cage of a MitoPark or control mice. Direct contact was allowed during the first 5 min during which MitoPark/control mouse was attacked typically 3–4 times. During the last 5 min of each trial, CD‐1 mouse was isolated within a rectangular wire‐mesh box to prevent further physical attacks without preventing olfactory, visual, and auditory contacts. At the end of SD, CD‐1 male was removed from the cage and any of their feces were also removed. MitoPark/control mice were left undisturbed for recovery. The positively‐charged SD protocol was consistent with introducing a virgin C57BL/6J female mouse to MitoPark/control male mice during the 4 h SD. 4–5 months old females that were group‐housed (4–5 mice per cage) since weaning were used. The stage of the menstrual cycle of mice was not verified. At the start of each hour, C57BL/6J female mouse was introduced into the cage for free and direct contact with MitoPark/control mice for 30 min then retrieved for the remaining 30 min. This cycle was repeated four times during the 4 h SD experience. At the end of SD, any feces left by female mice were removed from the cage and MitoPark/control mice were left undisturbed for recovery.

### EEG/EMG Data Acquisition, Sleep/Wake States Determination, and Data Processing and Analysis

EEG/EMG signals were amplified and filtered (0.65 Hz high‐pass filter for EEG, 5–30 Hz band‐pass filter for EMG), digitized at a sampling rate of 100, 128, or 256 Hz, and recorded using Sleep analysis software (SLEEPSIGN for animals, kissei Comtec Co, Nagaro, Japan) or Spike 2 software (Cambridge electronic design Limited).

First, 10 s epochs of EEG/EMG signals were screened offline using Sleepsign software for automatic scoring. EEG/EMG signals were then re‐loaded, visually examined, and manually corrected when necessary. Classification of sleep/wake states was performed according to established criteria into 3 states; 1) wake; characterized by desynchronized, low‐amplitude, and high‐frequency EEG signals and increased EMG activity, 2) NREM sleep defined as a sleep state with synchronized, high amplitude and low frequency EEG signals with decreased EMG activity and finally 3) REM state with its characteristic high theta rhythms in the EEG signals and strongly reduced EMG activity.

To analyze spectral proprieties of EEG across different vigilance states, EEG signals were decomposed into time‐frequency domain using Fast Fourier transform at the frequency range of 0–50 Hz for all experiments except the valence related SD (Figure 7) for which a broader frequency range of 0–64 Hz was analyzed with a 0.5 Hz resolution. Segmentation of EEG power bands was performed as follows; Delta (0.75–4 Hz), low theta (3.5–7.5 Hz), high theta (8–11.5 Hz), and gamma (30–50 Hz).

EEG power spectra were analyzed by FFT in 10 s epoch. Normalization of power spectra was done by computing the percentage of each 0.5 Hz bin from the mean power of each individual animal during baseline day. This computation was done in a state dependent manner; the power of each bin was first averaged for each specific stage individually, then normalized as a group by calculating the percentage of each bin from the mean power of each animal during baseline day.

Because the percentage of sleep lost during valence‐related SD experiments (Figure 7) was slightly different in CD‐1‐exposed relative to female‐exposed MitoPark/control mice, SD responses were normalized as the ratio of recovery NREM sleep for each animal over sleep lost during SD. Sleep lost is defined as the difference between total NREM sleep during corresponding baseline day and total NREM sleep still displayed during SD experiment.

(1) $$\begin{array}{ccc} \mathrm{SD}\;\mathrm{response} & = & \left[\mathrm{Recovery}\;\mathrm{NREM}\;\mathrm{sleep}/\mathrm{Lost}\;\mathrm{NREM}\;\mathrm{sleep}\right]\\ & = & [\mathrm{Recovery}\;\mathrm{NREM}\;\mathrm{sleep}/(\mathrm{Baseline}\;\mathrm{NREM}\;\mathrm{sleep}\\ & & -\mathrm{SD}\;\mathrm{NREM}\;\mathrm{sleep})] \end{array}$$

### Immunohistochemistry

Under deep anesthesia, mice were perfused with saline and then with PFA (4%). Brains were collected and post‐fixed in 4% PFA at 4 °C for 24 h. Brains were then immersed in 20% sucrose for 2 days at 4 °C and then sectioned into 40 µm coronal slices with a freezing microtome (thermo Scientific, Cryostat Nx70, USA). Brain sections were incubated in 0.3% hydrogen peroxide and then overnight in a PBS solution containing 0.1% Triton X‐100 and rabbit anti c‐fos (1:10 000, Sigma Aldrich, USA), rabbit anti TH (1:1000, Sigma Aldrich, USA) or rabbit anti BMAL1 (1:500, ThermoFisher, USA) at 4 °C. Afterward, sections were washed three times (10 min each) in PBS. Sections were then incubated for 2 h in biotinylated antibody (1:1000, Jackson ImmunoResearch Laboratories). Sections were again washed with PBS solution three times (10 min each) and then treated with avidin‐biotin complex (1:1000, Vectastain ABC Elite kit, Vector laboratories) for 1 h. After another round of 3 successive washing by PBS (10 min each), staining was visualized by monitored reaction with 3,3′‐diaminobenzidine and 0.01% hydrogen peroxide. The reaction was subsequently stopped by rising sections four times with PBS (10 min each). Sections were then mounted on gelatinized slides, dried and dehydrated in increasing gradients of ethanol, cleared in toluene, and cover‐slipped with Depex.

### Quantification and Statistical Analysis

All data were represented as mean ± sem. Statistical analyses were done with SigmaStat software. Paired and unpaired *t*‐tests were used for single value comparisons. One‐way ANOVA was used to compare more than two groups. Two‐way repeated measures ANOVA was used to perform group comparisons with multiple measurements. Regression analysis and Spearman test were used for correlations. Data were considered to be statistically significant if *p* < 0.05. Sidak's post hoc correction was used to control for multiple comparisons where appropriate. Figures were prepared using Prism 6.01.

## Conflict of Interest

The authors declare no conflict of interest.

## Author Contributions

K.F. conceived and designed the project, performed all experiments, analyzed data, made figures, and wrote the manuscript. All authors revised and improved the manuscript. M.Y. and T.D. supervised all aspects of the project.

## Supporting information

Supporting information

## Data Availability

The data that support the findings of this study are available from the corresponding author upon reasonable request.
