# Supplementary material for: Mechanisms of Sleep/Wake Regulation under Hypodopaminergic State: Insights from MitoPark Mouse Model of Parkinson's Disease
Source: Adv Sci (Weinh). 2022 Dec 14;10(5):2203170. doi: 10.1002/advs.202203170 (PMC9929135; doi:10.1002/advs.202203170)
Supplement: Supplementary file 1 — Supporting information [file ADVS-10-2203170-s001.pdf]

ADVANCED SCIENCE
Open Access

Supporting Information

for *Adv. Sci.*, DOI 10.1002/advs.202203170

Mechanisms of Sleep/Wake Regulation under Hypodopaminergic State: Insights from MitoPark Mouse Model of Parkinson's Disease

Karim Fifel, Masashi Yanagisawa and Tom Deboer*

Supporting Information

Mechanisms of Sleep/Wake Regulation Under Hypodopaminergic State: Insights From MitoPark Mouse Model of Parkinson's Disease

Fifel Karim[1,2]*, Masashi Yanagisawa[1], Tom Deboer[2]

1. International Institute for Integrative Sleep Medicine (WPI-IIIS), University of Tsukuba, 1-1-1 Tennodai, Tsukuba, Ibaraki, 305-8575, Japan.
2. Department of Cell and Chemical Biology, Laboratory of Neurophysiology, Leiden University Medical Center, P.O. Box 9600, 2300 RC, Leiden, The Netherlands.

Correspondence: Fifel Karim, fifel-k@hotmail.com

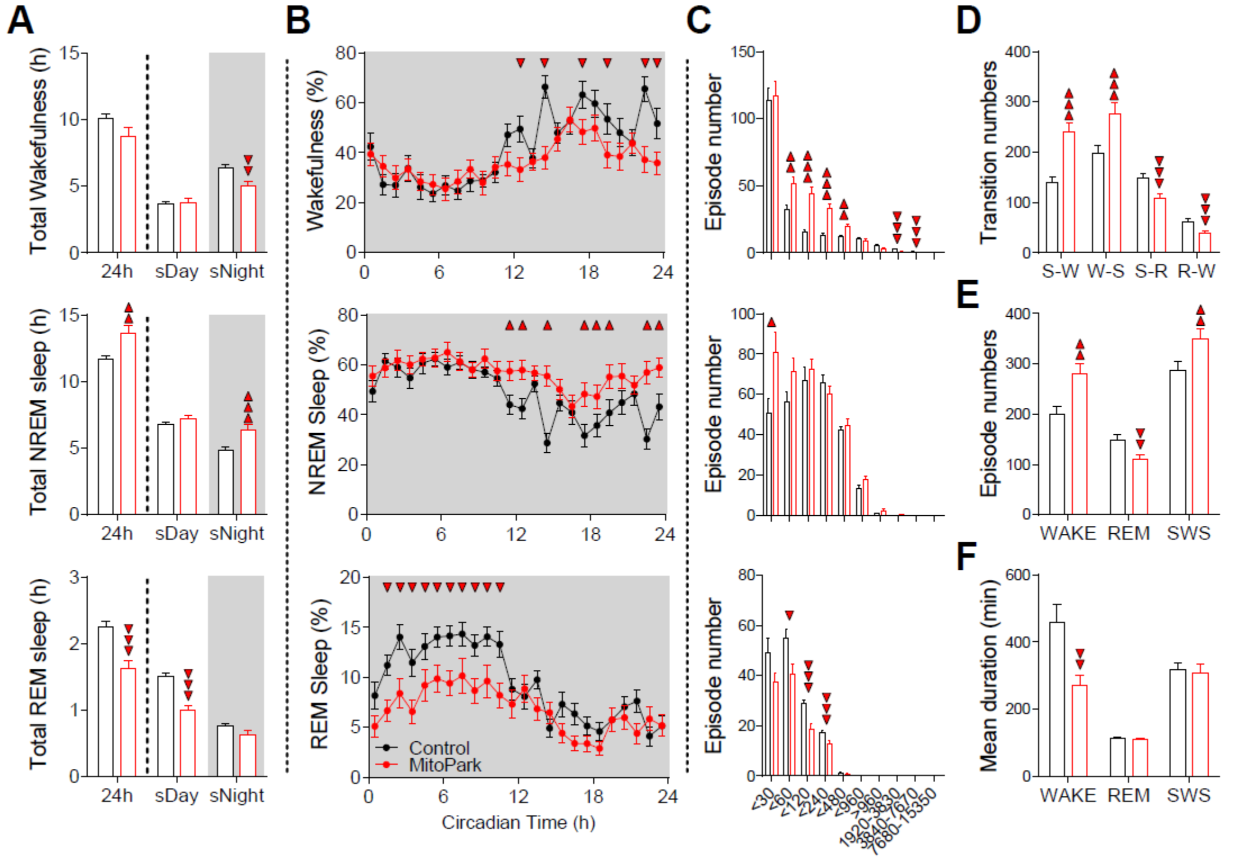

Figure S1. Sleep/wake architecture in MitoPark mice under constant DD conditions. Related to **Figure 1.** (**A**, **B**) Mean time (**A**) and hourly percentages (**B**) spent in wakefulness, NREM and REM sleep in control (n=19) and MitoPark mice (n=19). T test and Two-way ANOVA, $F_{1,23}$ (wake)=2.256, p<0.001; $F_{1,23}$ (NREM)= 2.019, p<0.003; $F_{1,23}$ (REM)=1.738, p=0.017. Sidak's post hoc analysis,▼ p<0.05,▼▼p<0,01,▼▼▼p<0,001. (**C**) Number of wake (up), NREM (middle) and REM sleep (down) bouts with different durations during the 24hr day (Two-way ANOVA, ▼ p<0.05, ▼▼ p<0,01, ▼▼▼ p<0,001). (**D**) Number of all vigilance states transitions is altered in MitoPark mice during LD (Two-way ANOVA followed by Sidak's post-hoc correction, ▼▼▼ p<0,001). (**E**) Episode number of both wake and NREM sleep was increased while the number of REM episodes decreased in MitoPark mice relative to control littermates (Two-way ANOVA followed by Sidak's post-hoc correction, ▼▼ p<0,01). (**F**) MitoPark mice had shorter wake episodes and normal durations of both REM and NREM sleep (Two-way ANOVA followed by Sidak's post-hoc correction,▼▼ p<0,01). Data represent mean ± sem.

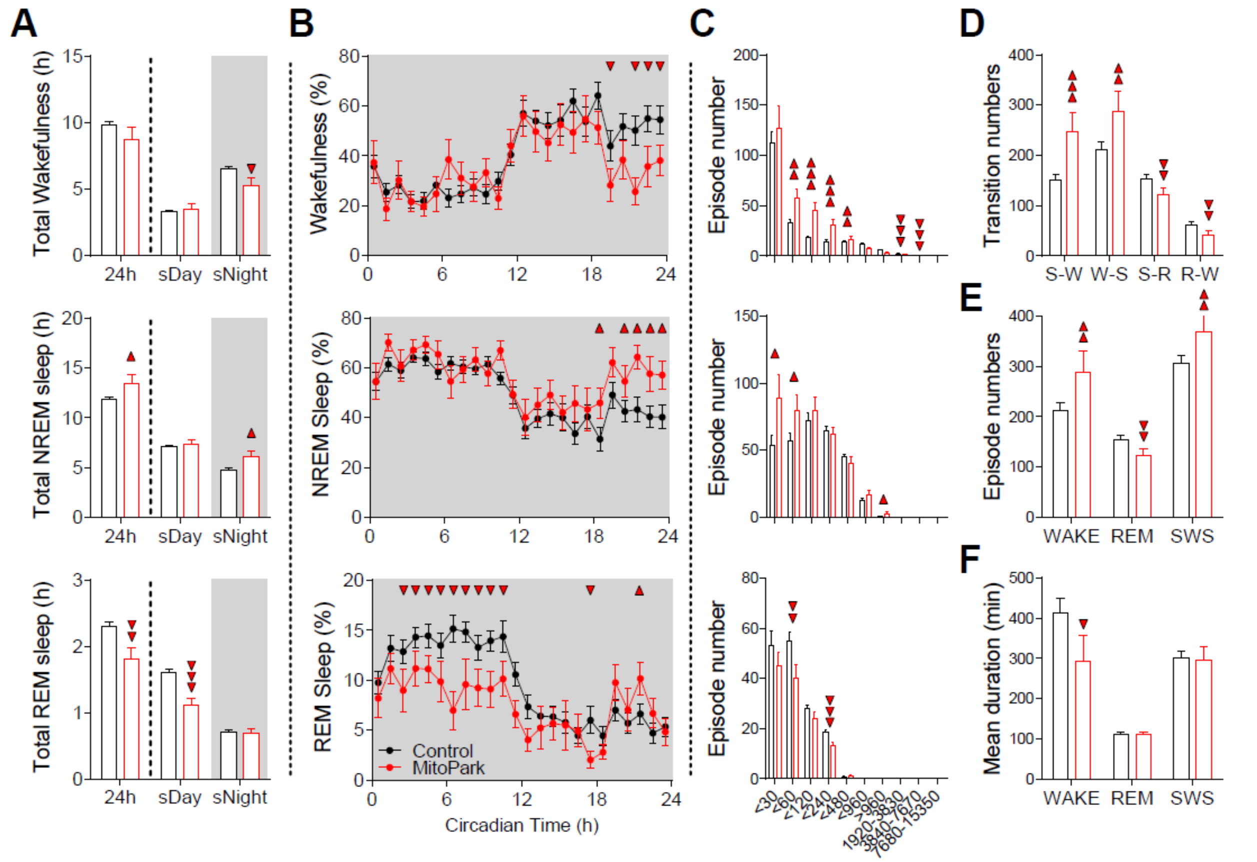

Figure S2. Sleep/wake architecture in early stages of Parkinsonism in MitoPark mice. Related to **Figure1**. (**A**, **B**) Mean time (**A**) and hourly percentages (**B**) spent in wakefulness, NREM and REM sleep in control (n=19) and MitoPark mice at the age of 4.8 ± 0.13 months (n=12). T test and Two-way ANOVA, $F_{1,23}$ (wake)=1.714, *p*=0.02; $F_{1,23}$ (NREM)= 1.491, *p*=0.065; $F_{1,23}$ (REM)=1.873, *p*=0.008. Sidak's post hoc analysis,▼ *p*<0.05, ▼▼*p*<0,01,▼▼▼*p*<0,001. (**C**) Number of wake (up), NREM (middle) and REM sleep (down) bouts with different durations during the 24hr day (Two-way ANOVA, 1 triangle *p*<0.05, 2 triangles *p*<0,01, 3 triangles *p*<0,001). (**D**) Number of all vigilance states transitions is altered in MitoPark mice during LD (Two-way ANOVA followed by Sidak's post-hoc correction, 2 triangles *p*<0,01, 3 triangles *p*<0,001). (**E**) Episode number of both wake and NREM sleep was increased while the number of REM episodes decreased in MitoPark mice relative to control littermates (Two-way ANOVA followed by Sidak's post-hoc correction, *p*<0,01). (**F**) MitoPark mice had shorter wake episodes and normal durations of both REM and NREM sleep (Two-way ANOVA followed by Sidak's post-hoc correction, *p*<0,05). Data represent mean ± sem.

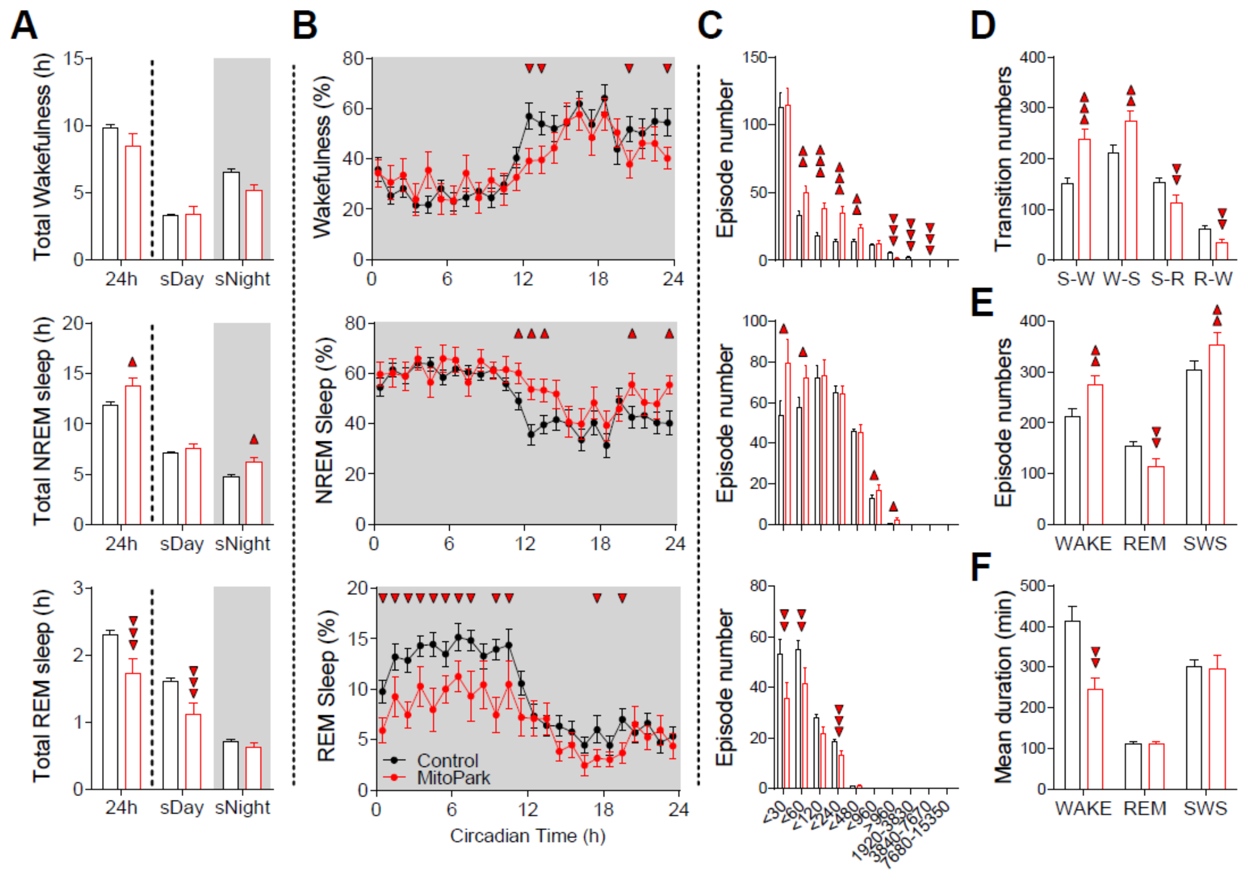

Figure S3. Sleep/wake architecture in advanced stages of Parkinsonism in MitoPark mice. Related to **Figure1**. (**A**, **B**) Mean time (**A**) and hourly percentages (**B**) spent in wakefulness, NREM and REM sleep in control (n=19) and MitoPark mice at the age of 6.4 ± 0.23 months (n=11). T test and Two-way ANOVA, $F_{1,23}$ (wake)=1.295, p<0.001; $F_{1,23}$ (NREM)= 1.33, p<0.001; $F_{1,23}$ (REM)=1.003, p<0.001. Sidak's post hoc analysis, ▼ p<0.05, ▼▼ p<0,01, ▼▼▼ p<0,001. (**C**) Number of wake (up), NREM (middle) and REM sleep (down) bouts with different durations during the 24hr day (Two-way ANOVA, 1 triangle p<0.05, 2 triangles p<0,01, 3 triangles p<0,001). (**D**) Number of all vigilance states transitions is altered in MitoPark mice during LD (Two-way ANOVA followed by Sidak's post-hoc correction, 2 triangles p<0,01, 3 triangles p<0,001). (**E**) Episode number of both wake and NREM sleep was increased while the number of REM episodes decreased in MitoPark mice relative to control littermates (Two-way ANOVA followed by Sidak's post-hoc correction, p<0,01). (**F**) MitoPark mice had shorter wake episodes and normal durations of both REM and NREM sleep (Two-way ANOVA followed by Sidak's post-hoc correction, p<0,01). Data represent mean ± sem. Data represent mean ± sem.

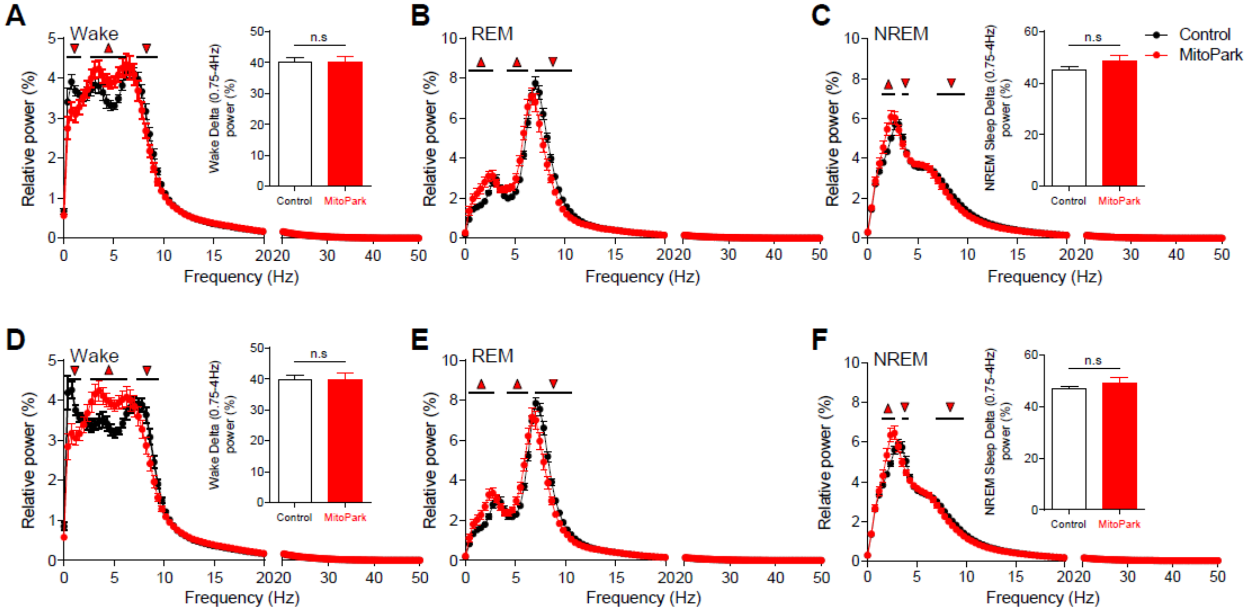

Figure S4. Qualitative changes of EEG spectral power during light and dark phases of the LD cycle. Related to **Figure 2.** (**A-F**) Power spectral density analysis of EEG during wake (**A, D**), REM (**B, E**) and NREM sleep (**C, F**) of the light (**A-C**) and the dark (**D-F**) phases of the LD cycle in control and MitoPark mice (Two-way ANOVA $p<0.001$; Sidak's post-hoc analysis, Triangle, $p<0.05$). Inserts in (**A**) and (**C**) show no significant difference in delta power between MitoPark mice and their littermate controls in both wake (**A**) and NREM sleep (**C**).

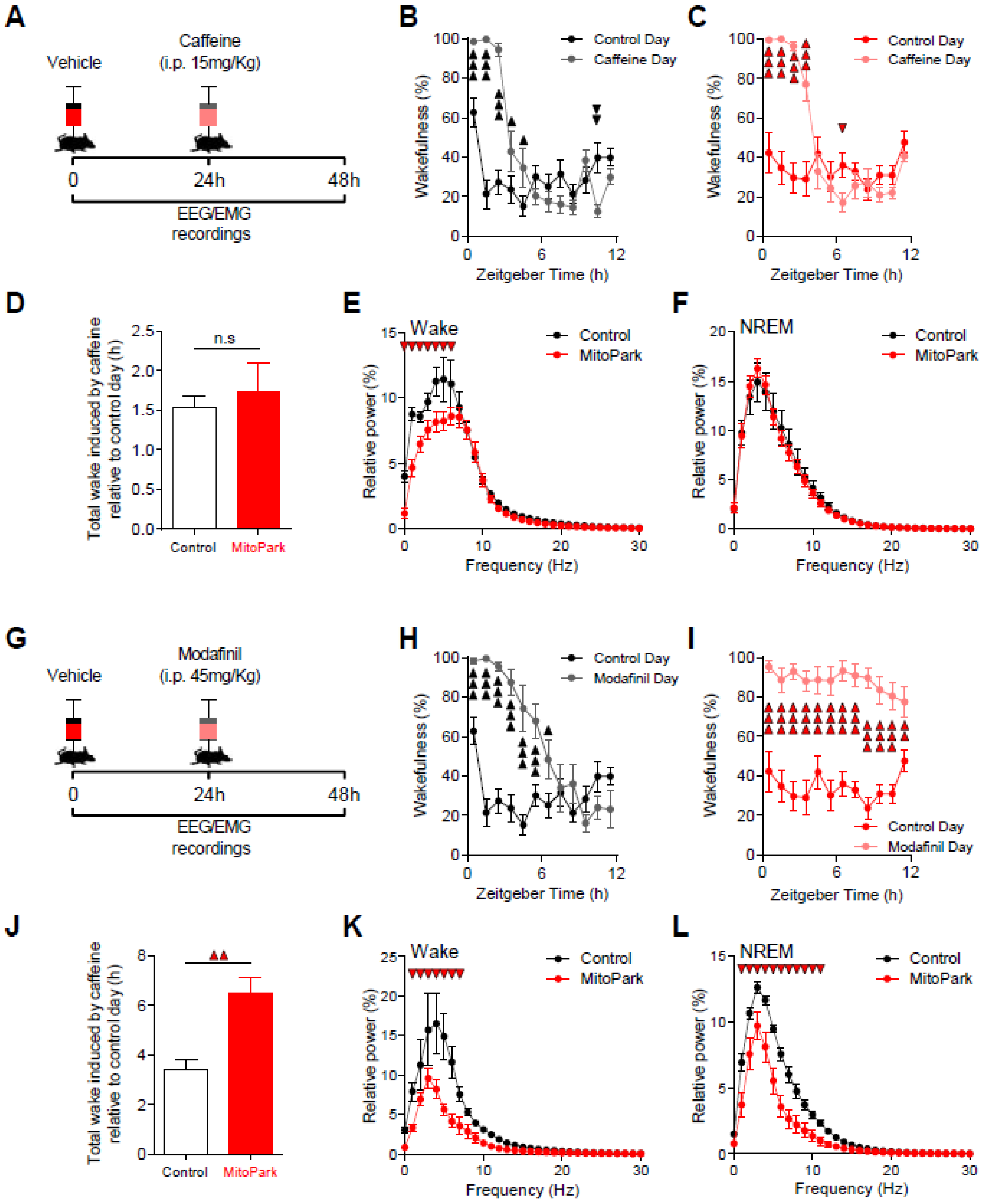

A
Vehicle
Caffeine
(i.p. 15mg/Kg)
0
24h
48h
EEG/EMG
recordings
B
Control Day
Caffeine Day
Wakefulness (%)
Zeitgeber Time (h)
C
Control Day
Caffeine Day
Wakefulness (%)
Zeitgeber Time (h)
D
Total wake induced by caffeine
relative to control day (h)
n.s
Control
MitoPark
E
Wake
Control
MitoPark
Relative power (%)
Frequency (Hz)
F
NREM
Control
MitoPark
Relative power (%)
Frequency (Hz)
G
Vehicle
Modafinil
(i.p. 45mg/Kg)
0
24h
48h
EEG/EMG
recordings
H
Control Day
Modafinil Day
Wakefulness (%)
Zeitgeber Time (h)
I
Control Day
Modafinil Day
Wakefulness (%)
Zeitgeber Time (h)
J
Total wake induced by caffeine
relative to control day (h)
Control
MitoPark
K
Wake
Control
MitoPark
Relative power (%)
Frequency (Hz)
L
NREM
Control
MitoPark
Relative power (%)
Frequency (Hz)

Figure S5. MitoPark mice have a normal response to caffeine but an exaggerated response to modafinil intake. Related to **Figure 2.** (**A**) Diagram showing the protocol of caffeine treatment (control, n=8; MitoPark, n=12). (**B**, **C**) Caffeine (15mg/Kg) significantly promoted wakefulness in both control (**B**) and MitoPark (**C**) mice (Two-way ANOVA revealed significant groups x time interaction $F_{1,11}$ (control)=16.196, p<0.001; $F_{1,11}$ (MitoPark)=14.735, p<0.001; Sidak's post hoc analysis,▼ p<0.05,▼▼p<0,01,▼▼▼p<0,001). (**D**) No difference in total wake induced during 4hrs following caffeine injection between MitoPark and control littermate mice (One-way ANOVA, $F_{1,18}$=0.184, p=0.673). (**E**, **F**) Power spectral density analysis of wake (**E**) and NREM sleep (**F**) EEG during caffeine injection day in MitoPark and littermate control mice (Two-way ANOVA revealed group x frequency interaction for wake but not NREM sleep, $F_{1,30}$ (wake)= 3.042, p<0.001; $F_{1,30}$ (NREM)= 0.253, p=1). (**G**) Diagram showing the protocol of modafinil treatment. (**H**, **I**) Modafinil (45mg/Kg) significantly promoted wakefulness in both control (**H**) and MitoPark mice (**I**) (Two-way ANOVA revealed significant groups x time interaction. $F_{1,11}$ (control)=11.677, p<0.001; $F_{1,11}$ (MitoPark)=1.019, p=0.43; Sidak's post hoc analysis,▼ p<0.05,▼▼p<0,01,▼▼▼p<0,001). (**J**) Total amount of wake induced during the 12hrs following modafinil treatment was significantly higher in MitoPark mice relative to their control littermates (One-way ANOVA, $F_{1,18}$=12.962, p=0.002). (**K**, **L**) Power spectral density analysis of wake (**K**) and NREM sleep (**L**) EEG during the day of modafinil treatment in MitoPark and control mice (Two-way ANOVA revealed group x frequency interaction. $F_{1,30}$ (wake)=4.321, p<0.001; $F_{1,30}$ (NREM)=4.517, p<0.001). Data represent mean ± sem.

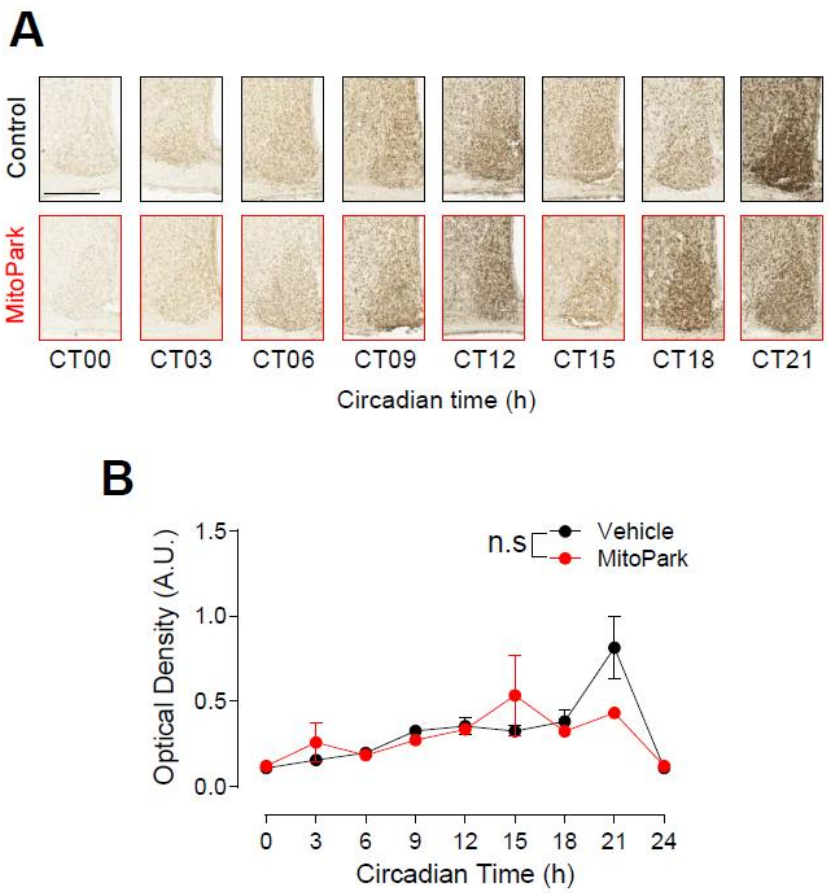

Figure S6. The circadian pattern of BMAL1 expression in the suprachiasmatic nucleus (SCN) of MitoPark mice is normal. (**A, B**) The circadian rhythm of BMAL1 expression in the SCN, as shown by immunohistochemistry, is not affected in MitoPark mice (n=3-4 per time point, Two-way ANOVA, $F_{1,7}$=1.706, p=0.15). Scale bar in (**A**) = 250 µm.

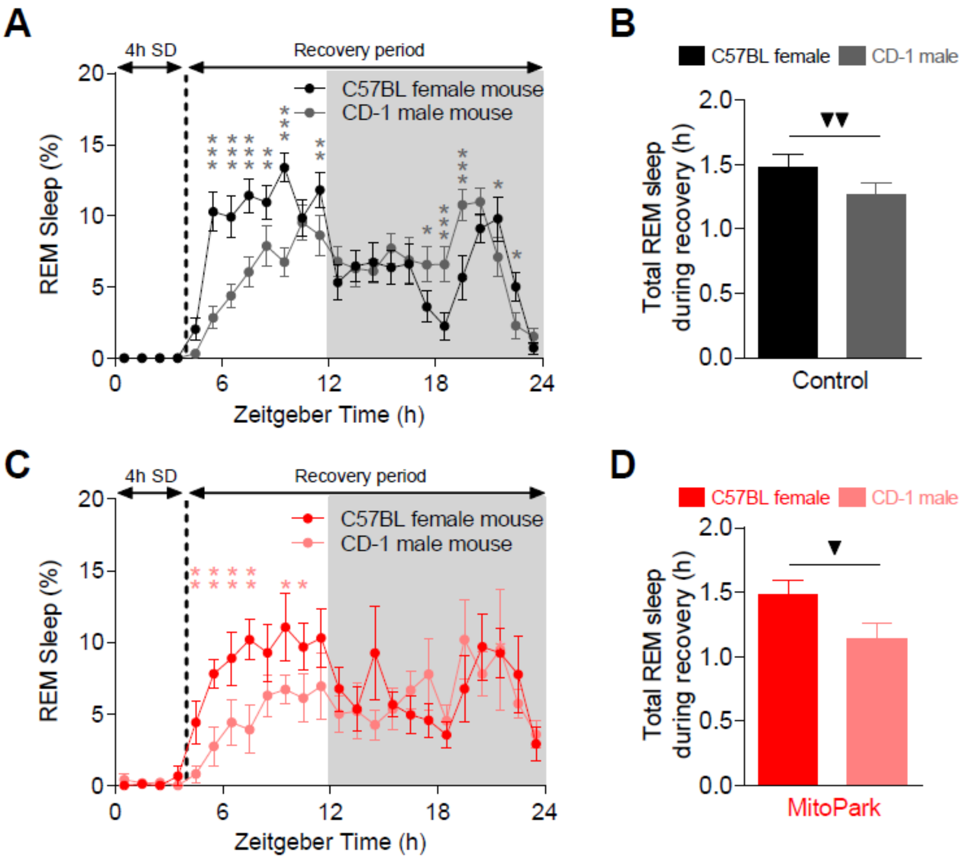

Figure S7. The modulation of REM sleep by motivational valence is not affected in MitoPark mice. Related to Figure 6. (**A, C**) Percentage of time spent in REM sleep in control (**A**) and MitoPark (**C**) mice during and after SD through the interaction with either CD-1 male or conspecific female (Two-way RM ANOVA revealed significant groups x time interactions. $F_{1,23}$ (control)= 7.286, *p*<0.001, $F_{1,23}$ (MitoPark)= 1.085, *p*=0.369, Sidak's post-hoc analysis, **p*<0.05, ***p*<0.01, ****p*<0.001). (**B**, **D**) Total time spent in REM sleep in control (**B**) and MitoPark mice (**D**) during recovery period (One-way RM ANOVA, $F_{1,16}$(control)=10.54, *p*=0.005; $F_{1,7}$(MitoPark)=7.126, *p*=0.037). Data represent mean ± sem.

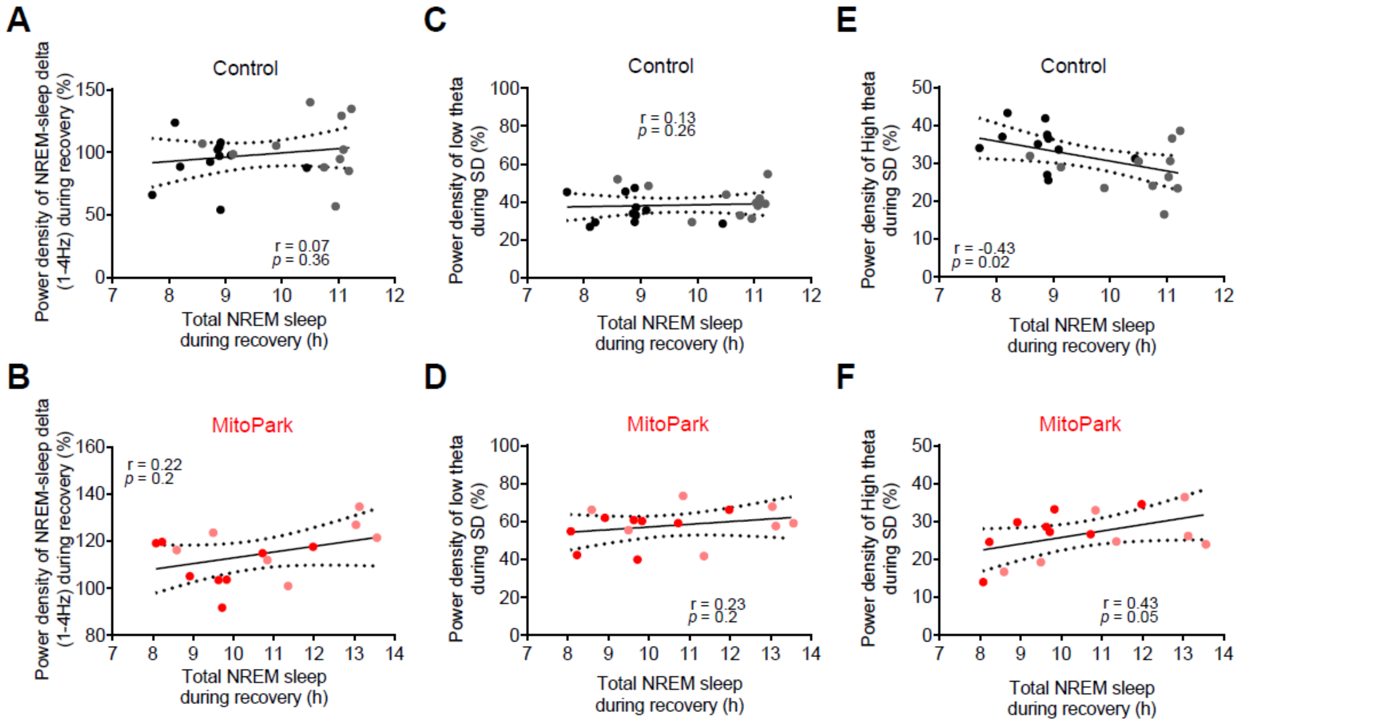

Figure S8. Correlations between EEG power densities and total amount of NREM sleep during recovery period. Related to Figure 7. (**A**, **B**) No significant correlation was found between NREM delta power and the amount of NREM sleep during recovery period after SD in both control (**A**) and MitoPark mice (**B**) (Spearman correlation). (**C**, **D**) The power density of low theta (3.5-7.5Hz) during SD correlated positively with the total amount of NREM sleep during recovery in control littermates (**C**) but not in MitoPark mice (**D**) (Spearman correlation). (**E**, **F**) The power density of high theta (8-11.5Hz) during SD correlated negatively with the total amount of NREM sleep during recovery in control littermates (**E**) but not in MitoPark mice (**F**) (Spearman correlation).

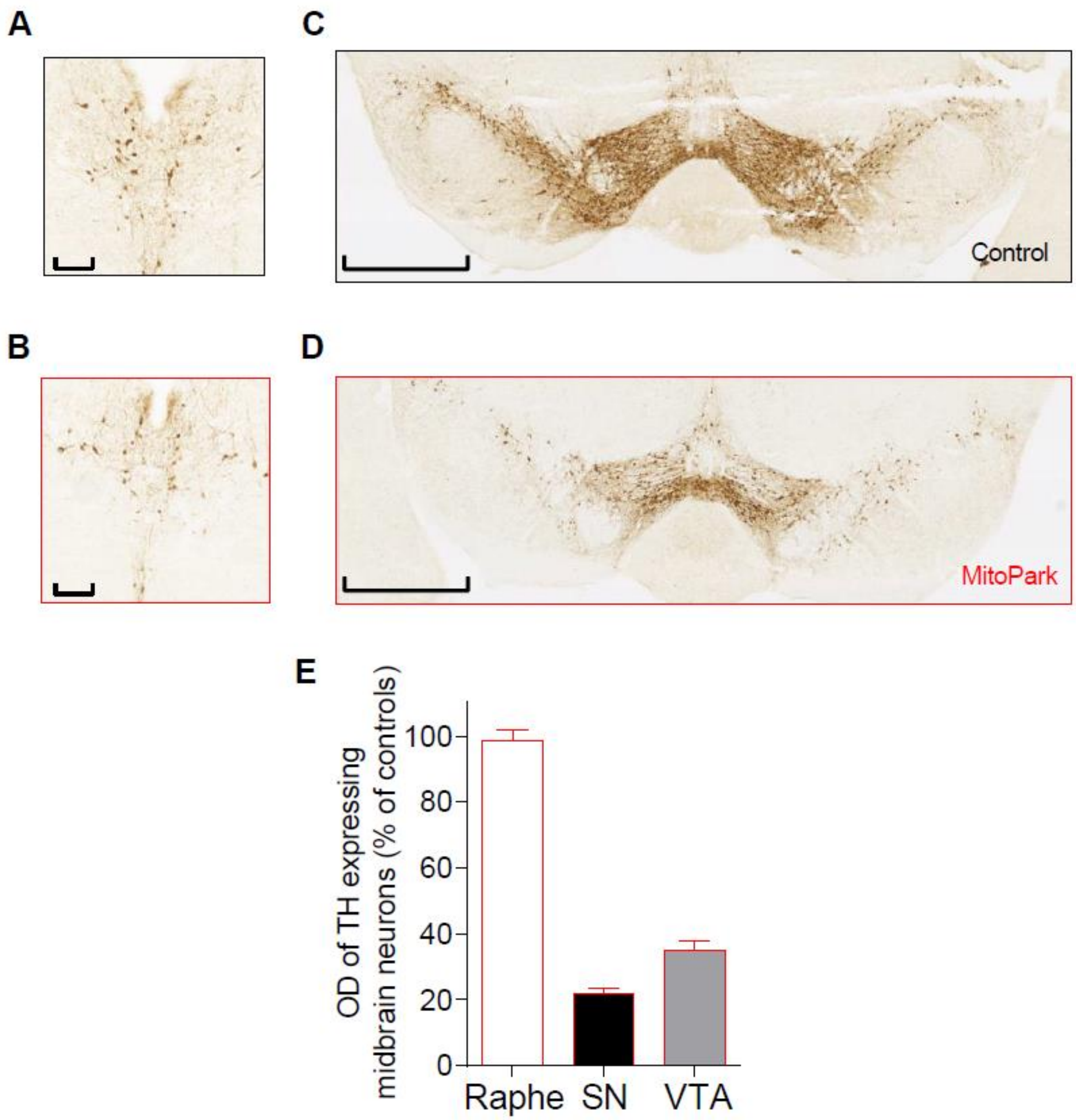

Figure S9. DA neurons in both Ventral tegmental area (VTA) and Substantia nigra (SN), but not in the Raphe, degenerate in MitoPark mice. (**A-D**) Tyrosine hydroxylase immunoreactivity in the raphe nuclei (**A**, **B**) and midbrain area (**C**, **D**) of control (**A**, **C**) and MitoPark (**B**, **D**) mice. (**C**) Semi-quantitative assessment of DA neurons loss in raphe (white bar), SN (black bar) and VTA (gray bar) of MitoPark mice relative to control littermates using optical density. Scale bar in (**A**, **B**) = 100 µm, in (**C**, **D**) = 1 mm. Data in (**C**) represent mean ± sem.
